# SOSSB1 and SOSSB2 mutually regulate protein stability through competitive binding of SOSSA

**DOI:** 10.1038/s41420-023-01619-3

**Published:** 2023-08-28

**Authors:** Qi Zhang, Rongjiao Hao, Hongxia Chen, Gangqiao Zhou

**Affiliations:** 1 Graduate Collaborative Training Base of Academy of Military Sciences, Hengyang Medical School, University of South China, Hengyang City, Hunan Province 421001 P.R. China; 2 School of Life Sciences, Hebei University, Baoding City, Hebei Province 071002 P.R. China; 3 State Key Laboratory of Proteomics, National Center for Protein Sciences at Beijing, Beijing Proteome Research Center, Beijing Institute of Radiation Medicine, Beijing, 100850 P.R. China; 4 Collaborative Innovation Center for Personalized Cancer Medicine, Center for Global Health, School of Public Health, Nanjing Medical University, Nanjing City, Jiangsu Province 211166 P.R. China

**Keywords:** Apoptosis, Homologous recombination

## Abstract

Human single-stranded DNA-binding protein homologs hSSB1 (SOSSB1) and hSSB2 (SOSSB2) make a vital impact on maintaining genome stability as the B subunits of the sensor of single-stranded DNA complex (SOSS). However, whether and how SOSSB1 and SOSSB2 modulate mutual expression is unclear. This study, demonstrated that the depletion of *SOSSB1* in cells enhances the stability of the SOSSB2 protein, and conversely, *SOSSB2* depletion enhances the stability of the SOSSB1 protein. The levels of SOSSB1 and SOSSB2 proteins are mutually regulated through their competitive binding with SOSSA which associates with the highly conservative OB-fold domain in SOSSB1 and SOSSB2. The destabilized SOSSB1 and SOSSB2 proteins can be degraded *via* the proteasome pathway. Additionally, the simultaneous loss of *SOSSB1* and *SOSSB2* aggravates homologous recombination (HR)-mediated DNA repair defects, enhances cellular radiosensitivity and promotes cell apoptosis. In conclusion, in this study, we showed that SOSSB1 and SOSSB2 positively regulate HR repair and the interaction between SOSSA and SOSSB1 or SOSSB2 prevents the degradation of SOSSB1 and SOSSB2 proteins via the proteasome pathway.

## Introduction

Single-stranded DNA (ssDNA)-binding protein (SSB/SSBP) has a high affinity to ssDNA, and participates as an auxiliary protein in DNA replication, recombination and repair [1]. The characteristic functional unit of SSBs is the oligonucleotide/oligosaccharide binding (OB) fold, which is a protein domain promoting the binding of SSBs to ssDNA [2]. Replication protein A (RPA) is the leading ssDNA-binding protein in eukaryotes and consists of three subunits, including RPA70, RPA32 and RPA14 (also known as RPA1, RPA2 and RPA3, separately) [3]. The three subunits of RPA form a highly stable complex, and OB-fold domains in each subunit interact with each other to allow the other parts of RPA protein to interact [4].

Besides encoding RPA, the human genome encodes two conserved SSB homologs, hSSB1 (SOSSB1) and hSSB2 (SOSSB2) [5], which are structurally similar, containing an N-terminal OB-fold domain and a conservative C-terminal domain [6]. The depletion of SOSSB1 or SOSSB2 causes in G1/S checkpoint defects, decrease HR repair efficiency, and increases radiosensitivity, which indicates that both proteins are involved in DNA repair [5]. SOSSB1 undergoes ubiquitination through Fbxl5 [7]. When DNA is damaged, SOSSB1 can be phosphorylated by ATM, acetylated by p300, or SUMOylated by PIAS2α. These three types of modifications inhibit the ubiquitin-mediated degradation of SOSSB1 and promote the recruitment of NBS1 by SOSSB1 at the site of DNA damage to respond to the damage [6]. Besides participating in DNA damage repair, SOSSB1 is also regulated the stability and transcriptional activity of p53 and p21 [8, 9]. SOSSB2 is needed for the recruitment of RPA after UV-induced DNA damage [10, 11]. However, only a few studies have investigated the function of SOSSB2.

The proteins SOSSB1 and SOSSB2 can form two separate heterotrimer complexes with SOSSA (also known as INTS3) and SOSSC (also known as C9orf80), respectively, which are called sensors of ssDNA complex 1 and 2 (SOSS1/2) [12]. The SOSS1/2 proteins participate in DNA damage repair (DDR). The most serious damage is to DNA involves double-strand breaks (DSBs) [13–15]. Cells use two major pathways repairing DSBs, containing NHEJ [16] and HR [17, 18]. The ssDNA produced during HR repair is susceptible to repair or degradation by nucleases. SOSS1 and SOSS2 complexes bind to ssDNA to prevent its degradation; however, only the B subunit of this proteins can directly bind to ssDNA [15, 19–21]. SOSSA binds to SOSSB1 and SOSSB2 to assemble the SOSS complex, but SOSSB1 and SOSSB2 do not directly interact with SOSSC [22]. The SSBP homologs SOSSB1 and SOSSB2 have an inverse relationship at the protein level.

This study, focused on investigating the mechanism and the role of the interaction between SOSSB1 and SOSSB2 in the SOSS complex. We found that SOSSB1 and SOSSB2 can regulate each other at the protein level through competitive binding to SOSSA. Although SOSSB1 and SOSSB2 have an inverse relationship at the protein level, they have a superimposed effect on DDR.

## Results

### The depletion of SOSSB1/SOSSB2 promoted the increase in the SOSSB2/SOSSB1 protein levels

**Fig. 1:**
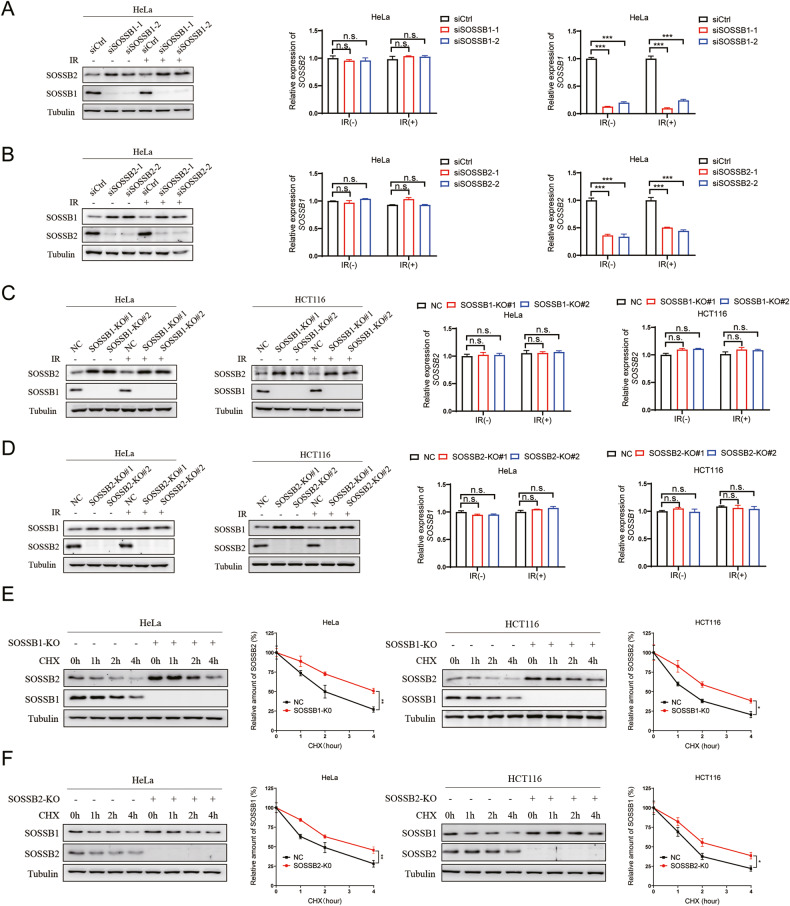
The depletion of SOSSB1/SOSSB2 promoted the increase in the SOSSB2/SOSSB1 protein levels. **A** Knockdown of *SOSSB1* upregulates the protein level of SOSSB2 before and after irradiation. HeLa cells transfected with the indicated siRNAs for 72 hours (h). Two hours after X-ray treatment (10 Gy), cells were subjected to Western blotting or real-time quantitative PCR (RT-qPCR) assays (n.s., not significant; ^***^P < 0.001; *n* = 3). **B** Knockdown of *SOSSB2* upregulates the protein level of SOSSB1 before and after irradiation. HeLa cells transfected with the indicated siRNAs for 72 h. Two hours after X-ray treatment (10 Gy), cells were subjected to Western blotting or RT-qPCR assays (n.s., not significant; ^***^P < 0.001; *n* = 3). **C** The deletion of *SOSSB1* upregulates the protein level of SOSSB2 before and after irradiation. *SOSSB1* were knocked out by CRISPR/Cas9 in HeLa and HCT116 cells. two hours after X-ray (10 Gy), cells were subjected to Western blotting or RT-qPCR assays (n.s., not significant; *n* = 3). **D** The deletion of SOSSB2 upregulates the protein level of SOSSB1 before and after irradiation. *SOSSB2* were knocked out by CRISPR/Cas9 in HeLa and HCT116 cells. Two hours after X-ray (10 Gy), cells were subjected to Western blotting or RT-qPCR assays (n.s., not significant; *n* = 3). **E** Deletion of SOSSB1 prolongs the protein half-life of SOSSB2. *SOSSB1*-knocked-out HeLa and HCT116 cells were incubated with 20 mg/mL cycloheximide (CHX) for the indicated periods of time. Lysates were harvested from the cells and explored by Western blotting assays (left pane). Quantitation of SOSSB2 protein were indicated in right pane (^**^P < 0.01; ^*^P < 0.05; *n* = 3). **F** Deletion of SOSSB2 prolongs the protein half-life of SOSSB1. *SOSSB2*-knocked-out HeLa and HCT116 cells were incubated with 20 mg/mL CHX for the indicated periods of time. Lysates were harvested from the cells and explored through Western blotting assays (left pane). Quantitation of SOSSB1 protein were indicated in right pane (n.s., not significant; ^**^P < 0.01; ^*^P < 0.05; *n* = 3).

The SOSS1 and SOSS2 complexes contain the same A subunit (SOSSA) and C subunit (SOSSC), but have different B subunits (SOSSB1 and SOSSB2), which indicates that SOSSB1 and SOSSB2 have complementary effects on the DNA damage response pathway [12]. Therefore, we investigated whether SOSSB1 and SOSSB2 have an inverse relationship. First, we knocked down *SOSSB1* in HeLa cells, finding that the SOSSB2 protein level elevated (Fig. 1A). However, by conducting quantitative PCR (qPCR) assays, we found that knocking down *SOSSB1* did not affect the mRNA levels of *SOSSB2* (Fig. 1A). Similarly, knocking down *SOSSB2* significantly enhanced the SOSSB1 protein levels, but it did not affect the *SOSSB1* mRNA levels (Fig. 1B). To validate our findings, we constructed knockout-stable transformation strains of *SOSSB1* and *SOSSB2* in HeLa and HCT116 cell lines. The results showed that knocking out *SOSSB1* increased the SOSSB2 protein levels, but did not affect *SOSSB2* mRNA levels in HeLa and HCT116 cells (Fig. 1C). Similarly, the *SOSSB2*-knocked-out cells had higher SOSSB1protein levels than the control HeLa and HCT116 cells, but their *SOSSB1* mRNA levels were similar (Fig. 1D). These finding indicated that the protein levels of SOSSB1 and SOSSB2 had an inverse relationship but the transcription of their respective genes was not affected.

We next investigated the protein stability of SOSSB2 in SOSSB1-knockdown HeLa and HCT116 cells and the protein stability of SOSSB1 in SOSSB2-knockdown HeLa and HCT116 cells. The results showed that knocking out *SOSSB1* prolonged the half-life of the SOSSB2 protein (Fig. 1E). Similarly, the SOSSB1 protein was more stable in the *SOSSB2*-knocked-out cells than in the control HeLa and HCT116 cells (Fig. 1F). Overall, our findings suggested that the depletion of SOSSB1 promotes the increase in the SOSSB2 protein by increasing the stability of the SOSSB2 protein, and vice versa.

### Competitive binding of SOSSB1 and SOSSB2 to SOSSA affected the protein levels of SOSSB1 and SOSSB2

**Fig. 2:**
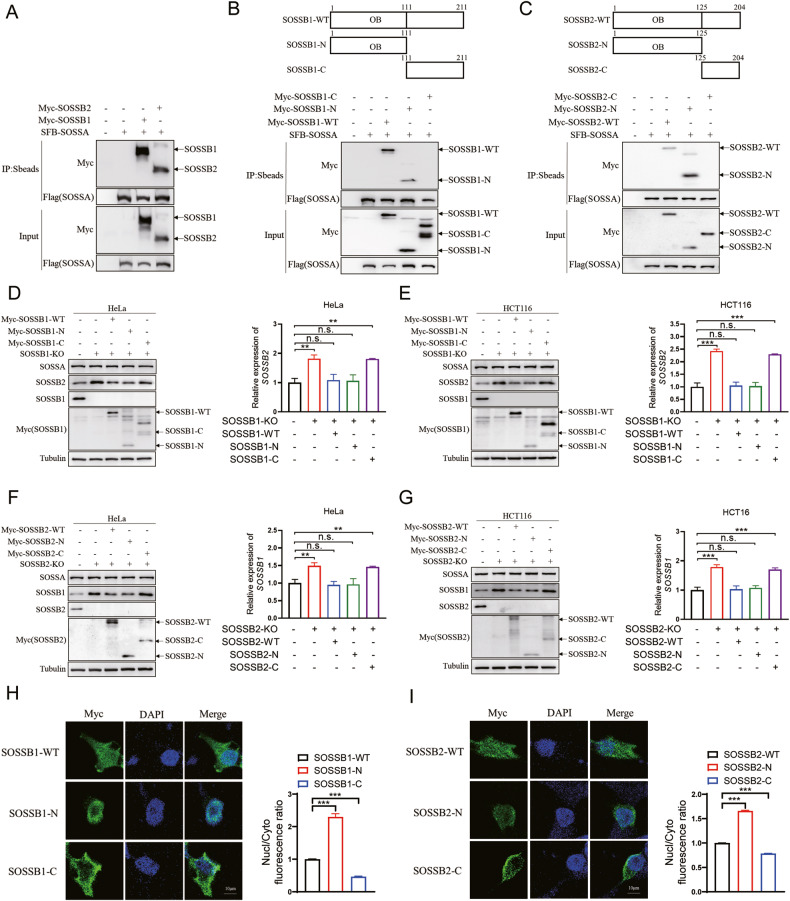
Competitive binding of SOSSB1 and SOSSB2 to SOSSA affected the protein levels of SOSSB1 and SOSSB2. **A** SOSSA interacts with SOSSB1 and SOSSB2. The 293T cells were subject to transfection with the indicated plasmids, and lysed after 24 hours (h). Cell lysates were analyzed by Western blotting or Co-immunoprecipitation (Co-IP) using S protein beads followed by Western blotting assays, as indicated. **B** The N-terminal oligosaccharide-binding fold (OB-fold) domain of SOSSB1 (a.a. 1–111, designated as SOSSB1-N) binds to SOSSA. Upper: Schematic description of the domains of SOSSB1. Lower: 293T cells co-transfected with SFB-SOSSA with various SOSSB1 mutants as indicated for 24 h were lysed in NETN buffer, and cell lysates were analyzed as **A**. **C** The N-terminal OB-fold domain of SOSSB2 (a.a. 1–125, designated as SOSSB2-N) binds to SOSSA. Upper: Schematic description of the domains of SOSSB2. Lower: 293T cells co-transfected with SFB-SOSSA with various SOSSB2 mutants as indicated for 24 h were lysed in NETN buffer, and cell lysates were explored as **A**. **D**, **E** SOSSB1 reduces the protein level of SOSSB2 through competitive binding with SOSSA. SOSSB1 mutants were transfected in SOSSB1-depleted HeLa (**D**) and HCT116 cells (**E**) for 24 h. Cells were subjected to Western blotting (left pane). Quantitation of SOSSB2 protein levels were shown in right pane (n.s., not significant; ^**^P < 0.01; ^***^P < 0.001; *n* = 3). **F**, **G** SOSSB2 reduces the protein level of SOSSB1 through competitive binding with SOSSA. SOSSB2 mutants were transfected in SOSSB2-depleted HeLa (**F**) and HCT116 cells (**G**) for 24 h. Cells were subjected to Western blotting (left pane). Quantitation of SOSSB1 protein levels were shown in right pane (n.s., not significant; ^**^P < 0.01; ^***^P < 0.001; *n* = 3). **H** The OB-fold domain is essential for the nuclear localization of SOSSB1. 293T cells transfected with various SOSSB1 mutants as indicated for 24h, were fixed with paraformaldehyde solution and stained using the indicated antibody and DAPI. Quantification of nuclear SOSSB1 percentage is presented as mean ± standard deviation (SD) (^***^P < 0.001; *n* = 3). **I** The OB-fold domain is essential for the nuclear localization of SOSSB2. 293T cells transfected with various SOSSB2 mutants as indicated for 24 h, were fixed with paraformaldehyde solution and stained using the indicated antibody and DAPI. Quantification of nuclear SOSSB2 percentage is presented as mean ± SD (^***^P < 0.001; *n* = 3).

The SOSSB1 and SOSSB2 proteins do not interact directly [12]. However, SOSSA can interact with both SOSSB1 and SOSSB2 and might help stabilize SOSSB1 and SOSSB2 in the cells. Thus, we hypothesized that the mutual influence between the SOSSB1 and SOSSB2 protein levels might be attributed to their respective interaction with SOSSA. The results of the co-immunoprecipitation (Co-IP) assays demonstrated that SOSSA made strongly interaction with SOSSB1 and SOSSB2 (Fig. 2A). We then identified the protein region(s) that mediate the interaction between SOSSB1 and SOSSA. Based on the conserved domain in SOSSB1, we constructed several deletion mutants of SOSSB1. The results of the Co-IP assays indicated that the N-terminal OB-fold domain of SOSSB1 (a.a. 1–111, designated as SOSSB1-N) binds to SOSSA, while the C-terminus of SOSSB1 (a.a. 112–211, designated as SOSSB1-C) fails to interact with SOSSA (Fig. 2B). We also investigate the interaction region between SOSSB2 and SOSSA. According to the conserved domains, SOSSB2 was classified into 2 different segments, the N-terminal OB-fold domain (a.a. 1–125, designated as SOSSB2-N) and C-terminal region (a.a. 125–204, designated as SOSSB2-C). We found that the N-terminal region of SOSSB2 is able to bind to SOSSA (Fig. 2C). Together, these results suggest that the OB-fold domains of SOSSB1 and SOSSB2 are needed for their bindings to SOSSA.

We then investigated whether the binding of SOSSA to SOSSB1 and SOSSB2 affects the SOSSB2 and SOSSB1 protein levels. We overexpressed the wild-type and mutant SOSSB1 in SOSSB1-deficient cells. WB assays showed that overexpression of wild-type SOSSB1 in SOSSB1-deficient HeLa and HCT116 cells can abolish the upregulation of the protein levels of SOSSB2 by SOSSB1 depletion (Fig. 2D, E). Overexpression the of OB-fold domain in SOSSB1 (i.e., SOSSB1-N) had an effect similar to wild-type SOSSB1, whereas, the overexpression of the SOSSB1 mutant that could not interact with SOSSA (i.e., SOSSB1-C) could not prevent the increase in the SOSSB2 protein levels by the depletion of SOSSB1 in SOSSB1-deficient cells (Fig. 2D, E). We also found that overexpressing the wild-type SOSSB2 protein and its OB-fold domain (i.e., SOSSB2-N) in SOSSB2-deficient HeLa and HCT116 cells prevented the increase in the SOSSB1 protein, whereas, overexpressing the SOSSB2 mutant protein that could not interact with SOSSA (i.e., SOSSB2-C) did not exhibit this effect in *SOSSB2*-deficient cells (Fig. 2F, G). We also determined whether the depletion of SOSSB1 and SOSSB2 can impair the expression of SOSSA. Our results showed that the SOSSA protein levels in SOSSB1 and SOSSB2-depleted cells were not different from those in control cells, whereas the deletion of SOSSA decreased the SOSSB1 and SOSSB2 protein levels (Figs. S1 and S2). Overall, these results indicated that SOSSB1 and SOSSB2 might reduce the protein levels of each other by competitively associating with SOSSA.

A study showed that SOSSA can promote the nuclear localization of SOSSB1 [19]. Similar to the findings of that study, we found that the SOSSB1 mutant that could not interact with SOSSA (SOSSB1-C) dispersed into the cytoplasm 0f HeLa cells, whereas, the SOSSB1 mutant containing the OB-fold domain (SOSSB1-N) accumulated in the nucleus (Fig. 2H). We also determined whether the interaction of the OB-fold structural domain with SOSSA is necessary for the nuclear localization of SOSSB2. We found that wild-type SOSSB2 was distributed in the nucleus and cytoplasm of HeLa cells (Fig. 2I). However, when the OB-fold domain of SOSSB2 was deleted, SOSSB2 could not localize in the nucleus (Fig. 2I). Our findings indicated that, SOSSA could facilitate the nuclear localization of SOSSB1 and SOSSB2.

### The destabilized SOSSB1 and SOSSB2 proteins were degraded *via* the proteasome pathway

**Fig. 3:**
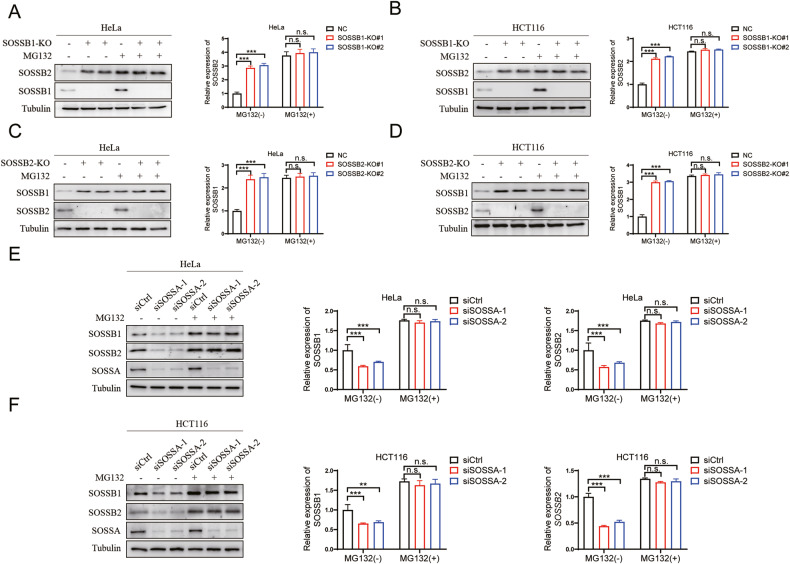
The destabilized SOSSB1 and SOSSB2 proteins were degraded *via* the proteasome pathway. **A**, **B** The destabilized SOSSB2 protein regulated by SOSSB1 was degraded by the ubiquitin proteasome pathway. HeLa **(A)** and HCT116 cells **(B)** with *SOSSB1* knockout were incubated with 20 µM MG132 for 4 hours (h), and probed for the indicated proteins by Western blotting assays (left pane). Quantitation of SOSSB2 protein levels were shown in right pane (n.s., not significant; ^***^P < 0.001; *n* = 3). **C**, **D** The destabilized SOSSB1 protein regulated by *SOSSB2* was degraded by the proteasome pathway. HeLa **(C)** and HCT116 cells **(D)** with *SOSSB2* knockout were incubated with 20 µM MG132 for 4 h, and probed for the indicated proteins by Western blotting (left pane). Quantitation of SOSSB1 protein levels were shown in right pane (n.s., not significant; ^***^P < 0.001; *n* = 3). **E**, **F** The destabilized SOSSB1 and SOSSB2 proteins regulated by SOSSA were degraded by the proteasome pathway. HeLa **(E)** and HCT116 cells **(F)** with *SOSSA* knockdown were incubated with 20 µM MG132 for 4 h, and probed for the indicated proteins by Western blotting (left pane). Quantitation of SOSSB1 and SOSSB2 protein levels were shown in right pane (n.s., not significant; ^**^P < 0.01; ^***^P < 0.001; *n* = 3).

According to the obtained findings, the depletion of SOSSB1/SOSSB2 elevated the protein levels, but not the mRNA levels, of SOSSB2/SOSSB1. To better understand these results, we investigated the underlying mechanism regulating the post-translational levels of SOSSB1 and SOSSB2 proteins. The stability of SOSSB1 can be regulated by the ubiquitin-proteasome system in HEK293T cells [7, 8]. Thus, we measured SOSSB1 protein levels in the presence of MG132, a specific ubiquitin-proteasome inhibitor. We found that treatment with MG132 in the absence of SOSSB1 did not further increase SOSSB2 protein levels (Fig. 3A, B). Similarly, in SOSSB2-deficient cells, treatment with MG132 did not further increase SOSSB1 protein levels (Fig. 3C, D). Based on these findings, the depletion of SOSSB2 prevented the degradation of SOSSB1 by the ubiquitin-proteasome and vice versa. As knocking down *SOSSA* destabilizes SOSSB1 and SOSSB2, we also investigated whether the reduction of SOSSB1 and SOSSB2 following the depletion of SOSSA was mediated by the proteasome system. We found that the downregulation of the SOSSB1 and SOSSB2 proteins induced by knocking down *SOSSA* was substantially changed by MG132 (Fig. 3E, F). There findings indicated that the proteasome pathway is crucial destabilizing SOSSB1 and SOSSB2.The destabilized SOSSB1 and SOSSB2 proteins influenced by the depletion of SOSSA might be mediated by the ubiquitin-proteasome pathway. However, the specific regulatory mechanism needs to be elucidated.

### The simultaneous depletion of SOSSB1 and SOSSB2 severely impaired HR repair and made the cells more sensitive to DNA damage

**Fig. 4:**
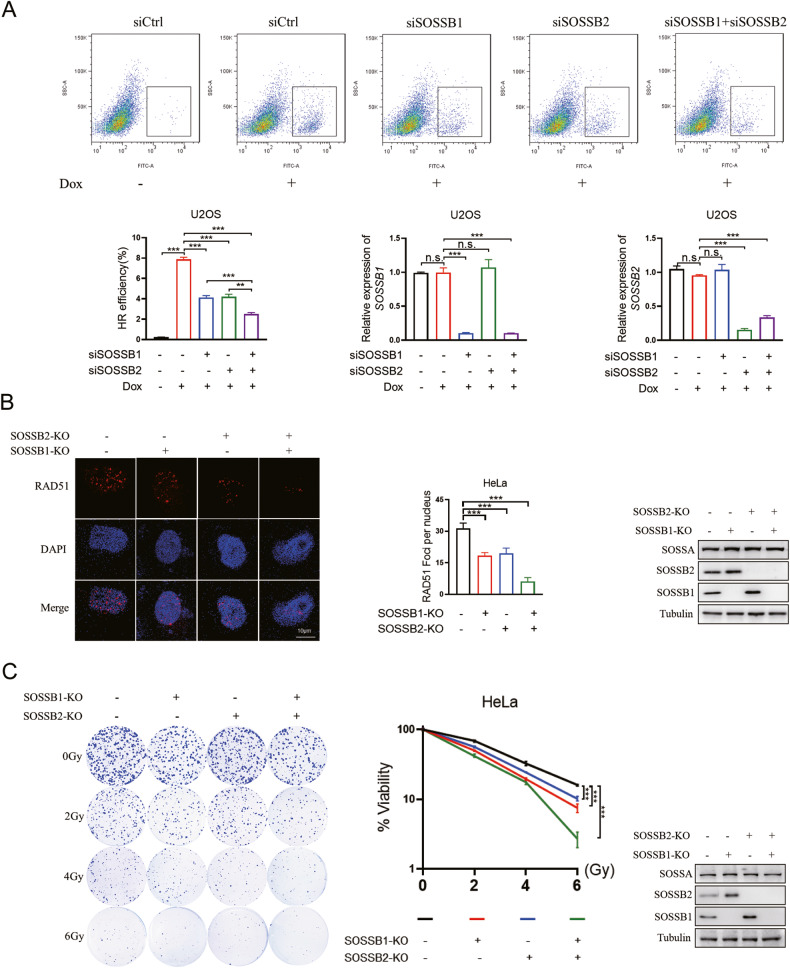
The Simultaneous depletion of SOSSB1 and SOSSB2 severely impaired HR repair and made the cells more sensitive to DNA damage. **A** Simultaneous deletion of *SOSSB1* and *SOSSB2* significantly reduces HR repair efficiency. U2OS cells with a single integration of DR-GFP were transfected twice with the indicated siRNA. Twenty-four hours (h) after the second transfection, cells were induced with doxycycline (Dox) and were subjected to flow cytometry analyses 48 h later (upper). Quantification of HR repair efficiency as well as mRNA expression levels are shown in the lower pane (n.s., not significant; ^***^P < 0.001; *n* = 3). **B** Simultaneous deletion of *SOSSB1* and *SOSSB2* significantly inhibits the recruitment of RAD51 at the sites of DNA damage. HeLa cells with *SOSSB1* and *SOSSB2* single and double knockout HeLa were treated with X-ray (10 Gy) and recovered for 2 h before fixation. The immunofluorescence assay was carried out as described in the methods. Left panel, the representative image of RAD51 foci; Right panel, the quantitative analysis of RAD51 foci and the identification of knockout effect by Western blotting assays (^***^P < 0.001; *n* = 10). **C** Simultaneous deletion of *SOSSB1* and *SOSSB2* significantly enhances ionizing radiation sensitivity. HeLa cells with *SOSSB1* and *SOSSB2* single and double knockout HeLa were treated with indicated doses of X-ray. Percentages of surviving colonies were determined 2 weeks later. Left panel, a representative image of colony formation; Right panel, the quantitative analysis of cell survival (n.s., not significant; ^***^P < 0.001; *n* = 3).

The SOSS complexes strongly influence maintenance of genome stability by regulating ionizing radiation (IR) sensitivity and HR repair. As SOSSB1 and SOSSB2 undergo mutual regulation in the SOSS complex, we investigated whether the double deletion of SOSSB1 and SOSSB2 can result in a greater DNA damage response compared to the depletion of either SOSSB1 or SOSSB2. First, we conducted gene conversion assays to determine the efficiency of HR repair using the DR-GFP reporter system. We found that the depletion of single *SOSSB1* or *SOSSB2* significantly impaired HR repair, whereas, the double knockdown of *SOSSB1* and *SOSSB2* resulted in a more serious defect in the efficiency of HR repair after treatment with doxycycline (Fig. 4A). The RAD51 protein is a vital component of the HR repair pathway. The formation of RAD51 foci is another indicator of the HR repair pathway. Thus, we performed immunofluorescence assays to examine the irradiation-induced RAD51 foci in single and double *SOSSB1-* and *SOSSB2-*knocked-out HeLa cells. Like to the findings of the gene conversion assay, the results of the immunofluorescence assays showed that X-ray-induced RAD51 foci formation was attenuated in single *SOSSB1-* or *SOSSB2-*knocked-out cells, and the loss of the RAD51 foci was more prominent when *SOSSB1* and *SOSSB2* were knocked out simultaneously (Fig. 4B). These results suggested that the simultaneous deletion of *SOSSB1* and *SOSSB2* contributed to a more serious defect in HR repair.

We treated SOSSB1- and SOSSB2-depleted HeLa cells with different doses of X-ray to measure their sensitivity to IR. Although the single SOSSB1- or SOSSB2-depleted cells had higher sensitivity to radiation than control cells, *SOSSB1* and *SOSSB2* double-knockout cells showed a more significant increase in sensitivity to IR than *SOSSB1* and *SOSSB2* single-knockout cells (Fig. 4C). We also found that apoptosis increased considerably in SOSSB1 or SOSSB2-deficient cells, and IR stress aggravated this increase in apoptosis. Simultaneously deleting SOSSB1 and SOSSB2 further increased the apoptosis of these cells (Fig. S3). These findings suggested that simultaneously deleting of SOSSB1 and SOSSB2 leads to a more serious HR repair defect and an increase in IR sensitivity and cell apoptosis.

## Discussion

In 2008, Richard et al. first described two other simple human SSB homologs, SOSSB1 (hSSB1) and SOSSB2 (hSSB2) [5]. Both SOSSB1 and SOSSB2 are involved in the formation of the sensor of the single-stranded DNA complex (SOSS). The SOSS complex is divided into two sub-complexes, including SOSS1 and SOSS2. Other researchers have found that SOSSA is the central adapter of the SOSS complex, and it closely interacts with SOSSB1, SOSSB2 and SOSSC [21]. In this study, we showed that SOSSB1 and SOSSB2 were competitively bound to SOSSA through their OB-fold domains, which affected the SOSSB1 and SOSSB2 protein levels. SOSSA also stabilized the SOSSB1 and SOSSB2 protein levels by inhibiting the proteasome degradation pathway, but SOSSB1 and SOSSB2 did not significantly affect the SOSSA protein levels. SOSSA was found to exert a key role in maintaining the expression of SOSSB1 and SOSSB2. Additionally, the nuclear localization of SOSSB1 and SOSSB2 was also impaired by the deletion of their OB-fold domains, which probably eliminated their ability to combine with SOSSA. However, the specific mechanism by which SOSSA affects the nuclear localization of SOSSB1 and SOSSB2 needs to investigated.

Several studies have shown that the ssDNA-binding proteins (SSBs/SSBPs) strongly influence eukaryotic DNA metabolism, not only by participating in DNA replication and DNA recombination but also by participating in various types of DNA damage repair [2, 23, 24]. RPA, is the most common SSB and is necessary for many DNA damage repair processes. During the process of HR repair, RAD51 replaces RPA with the help of a recombinant mediator and co-mediator to form a RAD51-ssDNA complex, which initiates the formation of RAD51 filaments [25, 26]. SOSSB1 is essential for efficiently repairing DSBs by the HR pathway [27]. SOSSB2 binds to chromatin and participates in ultraviolet (UV)-mediated DDR [10]. SOSS-depleted cells have high sensitivity to IR, G2/M checkpoint defects and impaired HR repair. The Mre11-NBS1-RAD50(MRN) complex can initiate the HR repair of DSBs [28]. It can also regulate the recruitment of SOSS (a sensor of ssDNA) [29]. In this study, we showed that SOSSB1 and SOSSB2 paly important roles in stimulating HR repair and resisting IR sensitivity. We found that the simultaneous depletion of SOSSB1 and SOSSB2 significantly decreased the recruitment of RAD51 to the DNA damage sites and resulted in accumulation of HR repair deficiency.

To summarize, in this study, we found that SOSSB1 and SOSSB2 mutually affected each other’s protein stability and levels through competitive binding with SOSSA. We found an inverse relationship at the protein level. The simultaneous deletion of SOSSB1 and SOSSB2 led to more serious effects on HR repair than single gene deletion.

## Materials and methods

### Antibodies

The anti-SOSSA, anti-SOSSB1, and anti-SOSSB2 antibodies were provided by Dr. Jun Huang (School of Zhejiang University, Hangzhou City, China). Anti-Flag (F1804) antibodies were purchased from Sigma. Anti-RAD51(11255-1-AP), Anti-Tubulin (66031-1-Ig) and Anti-Myc (60003-2-Ig) antibodies were purchased from Proteintech.

### Plasmid construction

The full-length and deletion mutants of *SOSSA*, *SOSSB1* and *SOSSB2* were amplified by PCR and subcloned into the pDONR201 vector by Gateway Technology (Invitrogen). The corresponding fragment in the entering vector was transferred to a Gateway-compatible target vector containing an N-terminal triple epitope tag (S-protein tag, flag epitope tag, and streptavidin-binding peptide tag) or a Myc epitope tag for expression in cells.

### Cell culture and transfection

The human cervical cancer cell HeLa, human colon cancer cell line HCT116 and human embryonic kidney cell line HEK293T were bought from the China Center for Type Culture Collection (CCTCC; Wuhan City, China). HeLa and HEK293T cells were kept in DMEM with the addition of 10% fetal bovine serum (FBS) and 1% penicillin-streptomycin solution. The HCT116 cells were maintained in RPMI 1640 medium with the supplement of 10% fetal bovine serum (FBS) and 1% penicillin-streptomycin solution. The cells were incubated under standard conditions (37 °C and 5% CO2) and 95% relative humidity. U2OS cells with DR-GFP integration were donated by Maria Jasin form the Memorial Sloan-Kettering Cancer Center (New York). All cell lines applied in the present were regularly checked via morphological observations and tested for mycoplasma contamination. In line with the manufacturer’s protocol, cell transfection was conducted with Lipofectamine 2000 (11668030, Invitrogen).

### RNA interference

All siRNA duplexes were purchased from RiboBio. The transfection of siRNAs was conducted using riboFECTTMCP (C10511-05, Ribobio) based on the manufacturer’s instructions. In the present study, the sequences of the siRNAs applied are presented in Table S1.

### Generation of stable cell lines

The *SOSSB1* and *SOSSB2* genes were knocked out in HeLa and HCT116 cell lines through the transfection of plasmids carrying a CRISPR/Cas9 system containing guide RNA sequences. The HeLa and HCT116 cells were transfected with 2 μg of plasmid with the use of Lipofectamine 2000 (11668030, Invitrogen). The medium was changed 6–8 hours (h) after transfection, and puromycin (2 μg/mL) was added for screening cells 24 h after the medium was altered. Then, the cells were maintained in a medium without puromycin. Single-cell colonies were generated by limiting dilution. Positive clones were isolated, and protein expression was determined via Western blotting assays.

### Exposure to radiation

Cells were irradiated in cell culture dishes at room temperature by an X-ray source at a dose rate of 119 cGy/min at the Beijing Institute of Radiation Medicine (Beijing, China). The cells were gathered 2 h after they were irradiated with 10 Gy of X-ray.

### RNA extraction and real-time PCR for gene expression

Total RNAs were extracted with RNApure Tissue & Cell Kit (CW0560, CWBIO) and reverse transcribed into complementary DNA (cDNA) with MonScript™ RTIII All-in-One Mix (MR05001M, Monad). Then, real-time quantitative PCR assays (RT-qPCR) were performed using a KAPA SYBR^®^ FAST Universal PCR kit (KK4601, KAPA Biosystems) and an ABI PRISM 7300 qPCR system (Applied Biosystems). Each sample was tested in triplicate. The final data were explored with the 2^-ΔΔCT^ method. Glyceraldehyde-3-phosphate dehydrogenase (GAPDH) was applied to be the internal reference. The primers applied in the present study are displayed in Table S1.

### Western blotting assays

Regarding protein analysis of whole-cell lysates, the cells were lysed in RIPA buffer, and a protease and phosphatase inhibitor cocktail (04693132001, Roche) for 15 minutes (min) on ice. Next, the cell lysates were gathered after centrifugation at 12,000 revolutions per minute (rpm) for 5 min at 4 °C. The proteins extracted from after cell lysis were electrophoresed in 1 × TGS electrophoresis buffer on SDS-PAGE gels and subsequently transferred onto PVDF (0.45 µm, Millipore) membranes. The membranes were blocked with 5% skim milk for 15 min and later, they were incubated with the indicated primary antibodies at 4 °C overnight, and anti-mouse (315-035-048, Jackson Immuno Research) or anti-rabbit (111-035-144, Jackson Immuno Research) secondary antibodies for 1 h at room temperature. In the end, the SuperSignal™ West Pico chemiluminescent substrate kit (34580, Thermo) and a Western blotting detection system (Bio-Rad) were used to detect the immunoreactive bands.

### Co-Immunoprecipitation (Co-IP) assays

We transfected HEK293T cells with overexpression or empty vector. The transfected cells were lysed and sonicated for 40 second (s) in NETN buffer (0.5% NP-40, 20 mM Tris-HCL [pH8.0], 0.1 mM NaCl, 1 mM EDTA, and protease inhibitor) for 15 min on ice. Additionally, cell lysates were collected after sonication by centrifugation at 12,000 rpm for 5 min at 4 °C. Then, 10% of the supernatant was taken as input. The remaining supernatant was used for IP by incubating with S protein beads (69704-2 ml, Millipore) for 4 h. The proteins were released from the beads by boiling after they were washed with NETN buffer, and the, they were analyzed via immunoblotting with the indicated antibodies.

### Protein stability assays

First, HeLa and HCT116 cells were incubated with 20 µg/mL cycloheximide (HY-12320, MCE) or 20 µM MG132 (HY-13259, MCE) for a certain duration. The cells were lysed in RIPA buffer including protease inhibitors for 15 min on ice. Next, the cell lysates were gathered after centrifugation at 12,000 rpm for 5 min at 4 °C. Via Western blotting assays and quantified by the ImageJ software, the SOSSB1 and SOSSB2 protein levels were estimated.

### Immunofluorescence assays

Cells cultured on coverslips were subject to treatment with 10 Gy of X-ray irradiation followed by recovery for 2 h. Next, the cells were then fixed with a 4% paraformaldehyde solution for 10 min at room temperature, and later, extracted for 5 min with a buffer including 0.5% Triton X-100. After washing, the samples were blocked with 5% horse serum and incubated with primary antibodies for 20 min at room temperature. The samples were washed and incubated with anti-mouse (FITC Rabbit anti Mouse IgG, 115-095-146, Jackson Immuno Research) secondary antibodies for 20 min at room temperature. Then, these samples were counterstained with DAPI to visualize nuclear DNA. The fluorescence intensity was analyzed using the ImageJ software.

### Cell survival assays

Cells (1 × 10^3^) were cultured in six-well plates in triplicates. After the cells were sub-cultured for 24 h, they were irradiated with X-ray (2, 4 and 6 Gy), and then, they were subject to incubation for 11 days. The colonies formed were fixed and stained with Coomassie blue. With the use of the ImageJ software, the number of colonies were counted. The findings were the averages of the data obtained from three independent experiments.

### Homologous recombination repair assays

A U2OS cell clone stably expressing the homologous recombination (HR) reporter DR-GFP was depicted in another study [30]. In total, 1 × 10^5^ cells were inoculated in six-well plates. One day after seeding, the U2OS cells with DR-GFP single integration were transfected with siRNAs targeting *SOSSB1* or *SOSSB2* or with non-targeted control siRNAs twice, with an interval of one day. After 24 h of the second transfection, the cells were treated with doxycycline (D8960, Solarbio) for 48 h to induce DSBs, and the repair frequency was calculated according to the percentage of GFP-positive cells through conducting flow cytometry assays.

### Cell apoptosis assays

Cells were inoculated in six-well plates. After the cells were inoculated for 24 h, they were irradiated with X-ray (10 Gy) and then, incubated for 24 h. The cells were digested with 0.25% trypsin and gathered after centrifugation at 1100 rpm for 5 min, and used (1 × 10^5^ cells) for antibody labeling in line with the instructions of the manufacturer. Based on the PE Annexin V Apoptosis Detection Kit I (559763, BD Biosciences), cell apoptosis was analyzed.

### Statistical analysis

All the date were indicated to be the mean ± standard deviation (SD). The differences were identified by conducting the analysis of variance (ANOVA) followed by the Tukey-Kramer multiple comparisons test or unpaired two-tailed Student’s *t* test with the application of the GraphPad 8 software. All differences among and between groups were regarded to be statistical significance at *P* < 0.05.

### Supplementary information

Supplementary Information

Original western blots

## Data Availability

All data generated or analyzed during this study are included in this published article and its Additional files. Additional data are available from the corresponding authors on reasonable request.
