# Supplementary material for: SOSSB1 and SOSSB2 mutually regulate protein stability through competitive binding of SOSSA
Source: Cell Death Discov. 2023 Aug 28;9:319. doi: 10.1038/s41420-023-01619-3 (PMC10462637; doi:10.1038/s41420-023-01619-3)
Supplement: Supplementary file 1 — Supplementary Information [file 41420_2023_1619_MOESM1_ESM.pdf]

**Supplementary Information**

# SOSSB1 and SOSSB2 mutually regulate protein stability through competitive binding of SOSSA

**Qi Zhang[1], Rongjiao Hao[2], Hongxia Chen[3]✉, and Gangqiao Zhou[1,2,3,4]✉**

[1]*Graduate Collaborative Training Base of Academy of Military Sciences, Hengyang Medical School, University of South China, Hengyang City, Hunan Province,421001, P.R. China;*

[2] *School of Life Sciences, Hebei University Baoding City, Hebei Province,071002, P.R. China;*

[3]*State Key Laboratory of Proteomics, National Center for Protein Sciences at Beijing, Beijing Proteome Research Center, Beijing Institute of Radiation Medicine, Beijing,100850, P.R. China;*

[4]*Collaborative Innovation Center for Personalized Cancer Medicine, Center for Global Health, School of Public Health, Nanjing Medical University, Nanjing City, Jiangsu Province,211166, P.R. China;*

✉Corresponding Author.

**Address correspondence to:**

Dr. Gangqiao Zhou, State Key Laboratory of Proteomics, National Center for Protein Sciences at Beijing, Beijing Proteome Research Center, Beijing Institute of Radiation

Medicine, 27 Taiping Road, Beijing 100850, P. R. China. E-mail: zhougq114@126.com; Phone: 86-10-66931201.

**or**

Dr. Hongxia Chen, State Key Laboratory of Proteomics, National Center for Protein Sciences at Beijing, Beijing Proteome Research Center, Beijing Institute of Radiation Medicine, 27 Taiping Road, Beijing 100850, P. R. China. E-mail: chenhongxia0626@163.com; Phone: 86-10-66930297.

**Supplementary information includes:**

**Supplementary figures**

Fig. S1. Knockdown of *SOSSB1* or *SOSSB2* has no significant influence on the protein level of SOSSA.

Fig. S2. SOSSB1 and SOSSB2 play no role in regulating the protein stability of SOSSA.

Fig. S3. Deletion of *SOSSB1* and *SOSSB2* promotes cell apoptosis.

**Supplementary table**

Table S1. siRNA and primer sequences.

**Supplementary figures**

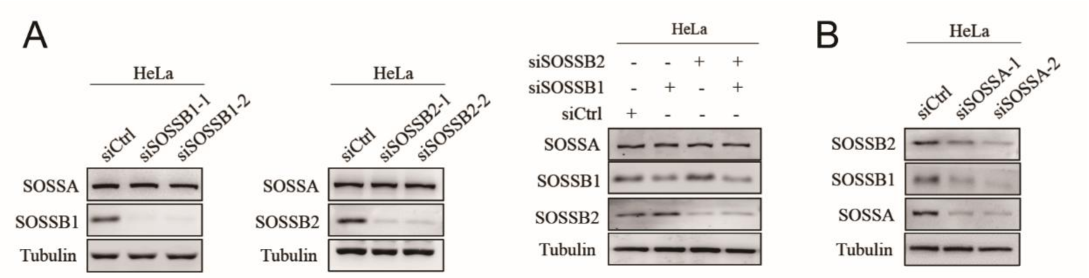


**Fig. S1 Knockdown of *SOSSB1* or *SOSSB2* has no significant influence on the protein level of SOSSA. A** Knockdown of *SOSSB1* or *SOSSB2* has no influence on the protein levels of SOSSA. HeLa cells transfected with the indicated siRNAs for 72 hours (h) were subjected to Western blotting assay. **B** Knockdown of *SOSSA* impairs the protein levels of SOSSB1 and SOSSB2. HeLa cells transfected with the indicated siRNAs for 72 h were subjected to Western blotting assays.

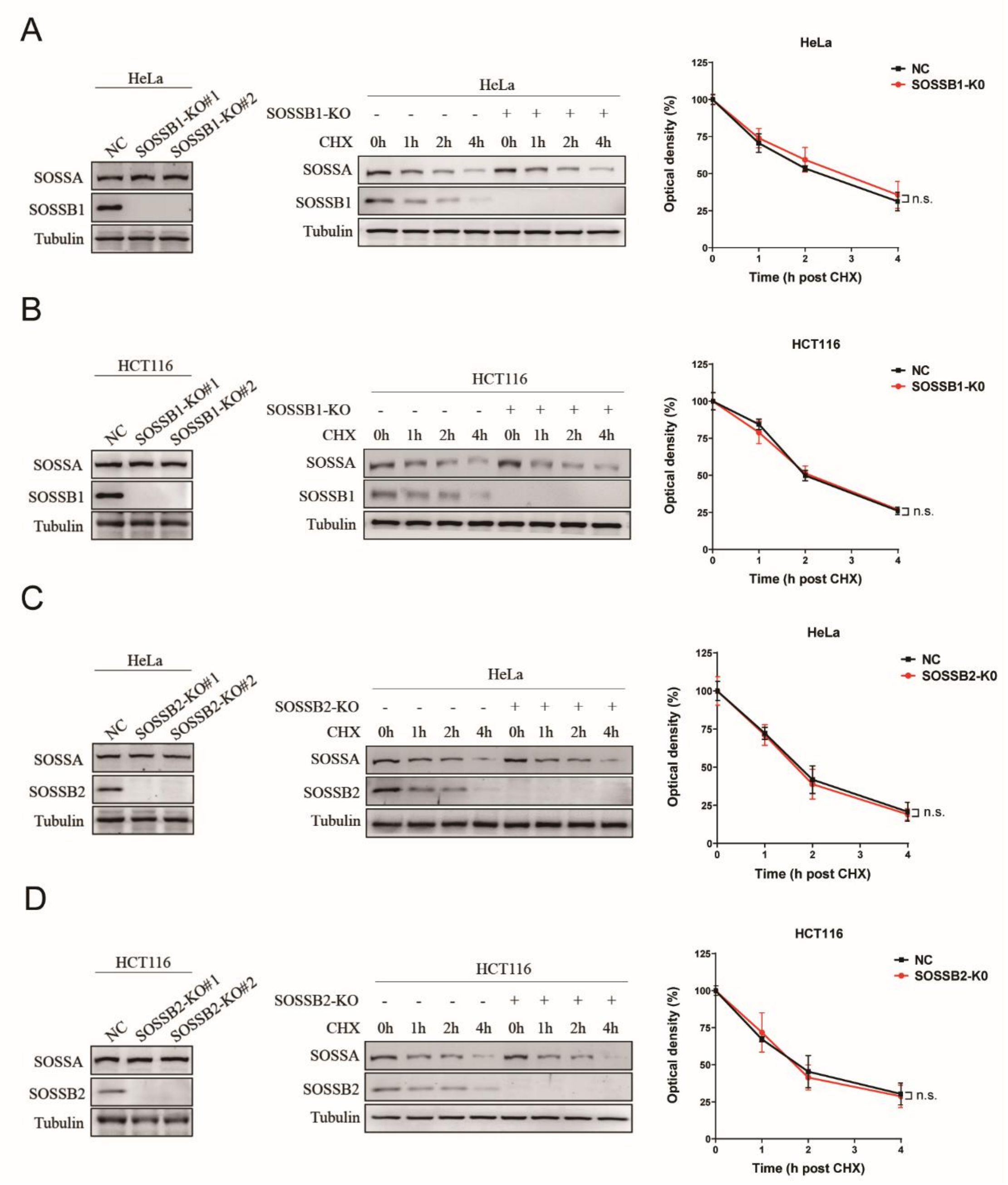


**Fig. S2 SOSSB1 and SOSSB2 play no role in regulating the protein stability of SOSSA. A, B** Knockout of *SOSSB1* does not impact the protein level and half-life of SOSSA. *SOSSB1*-knocked-out HeLa (A) or HCT116 (B) cells were incubated with 20 mg/mL cycloheximide (CHX) for the indicated periods of time. Lysates were harvested from the cells and analyzed by Western blotting assays (left panel). Quantitation of SOSSA protein levels were shown in right pane (n.s., not significant;

n = 3). **C, D** Knockout of *SOSSB2* does not impact the protein level and half-life of SOSSA. *SOSSB2*-knocked-out HeLa (C) and HCT116 cells (D) were incubated with 20 mg/mL CHX for the indicated periods of time. Lysates were harvested from the cells and analyzed by Western blotting assays (left pane). Quantitation of SOSSA protein levels were shown in right pane (n.s., not significant; n = 3).

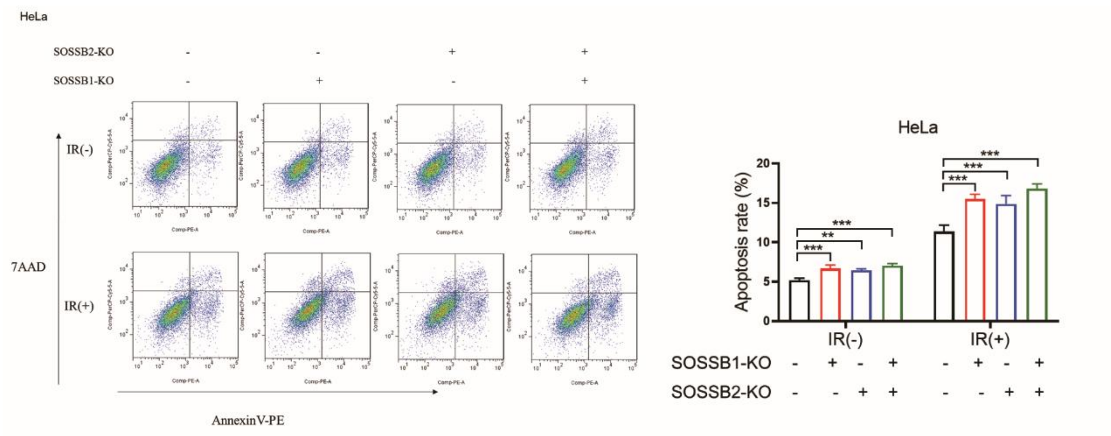


**Fig. S3 Deletion of *SOSSB1* and *SOSSB2* promotes cell apoptosis.** Simultaneous deletion of *SOSSB1* and *SOSSB2* significantly inhibits cell apoptosis. HeLa cells with *SOSSB1* and *SOSSB2* single and double knockout were exposed to 10 Gy of X-ray exposure. Cells were incubated for 24 hours (h) and then stained with phycoerythrin and 7-aminoactinomycin D antibodies. Apoptosis was assessed by flow cytometry analysis (left pane). The quantitation of apoptosis level is in the right pane ($^{***}P <$ 0.001; $^{**}P < 0.01$, n = 3).

**Table S1 siRNA and primer sequences.**

| Names/Genes | Sequences (5'→3' on minus strand) |
|---|---|
| siSOSSA#1 | GAUGAGAGUUGCUAUGACAdTdT |
| siSOSSA#2 | CCAAGCGAGCUGUGACGAAdTdT |
| siSOSSB1#1 | CGACGGGACCUUUGUGAAdTdT |
| siSOSSB1#2 | GCAGAAGAUUGGAGAAUUCdTdT |
| siSOSSB2#1 | CGUGCAAAGUAGCAGAUAAdTdT |
| siSOSSB2#2 | AGAGUGAACAGAAGAAUAAdTdT |
| GAPDH | Forward: CGACCACTTTGTCAAGCTCA |
| | Reverse: TTACTCCTTGGAGGCCATGT |
| SOSSB1 | Forward: TTCAGTGAGCCAAACCCAGA |
| | Reverse: GAAGCTGAAGGGTTGCTGTC |
| SOSSB2 | Forward: AAGACGGCCATGAAGTGAGA |
| | Reverse: TGGATGCATACCCTCTGGTC |
