# Supplementary material for: SOSSB1 and SOSSB2 mutually regulate protein stability through competitive binding of SOSSA
Source: Cell Death Discov. 2023 Aug 28;9:319. doi: 10.1038/s41420-023-01619-3 (PMC10462637; doi:10.1038/s41420-023-01619-3)

Figure-1

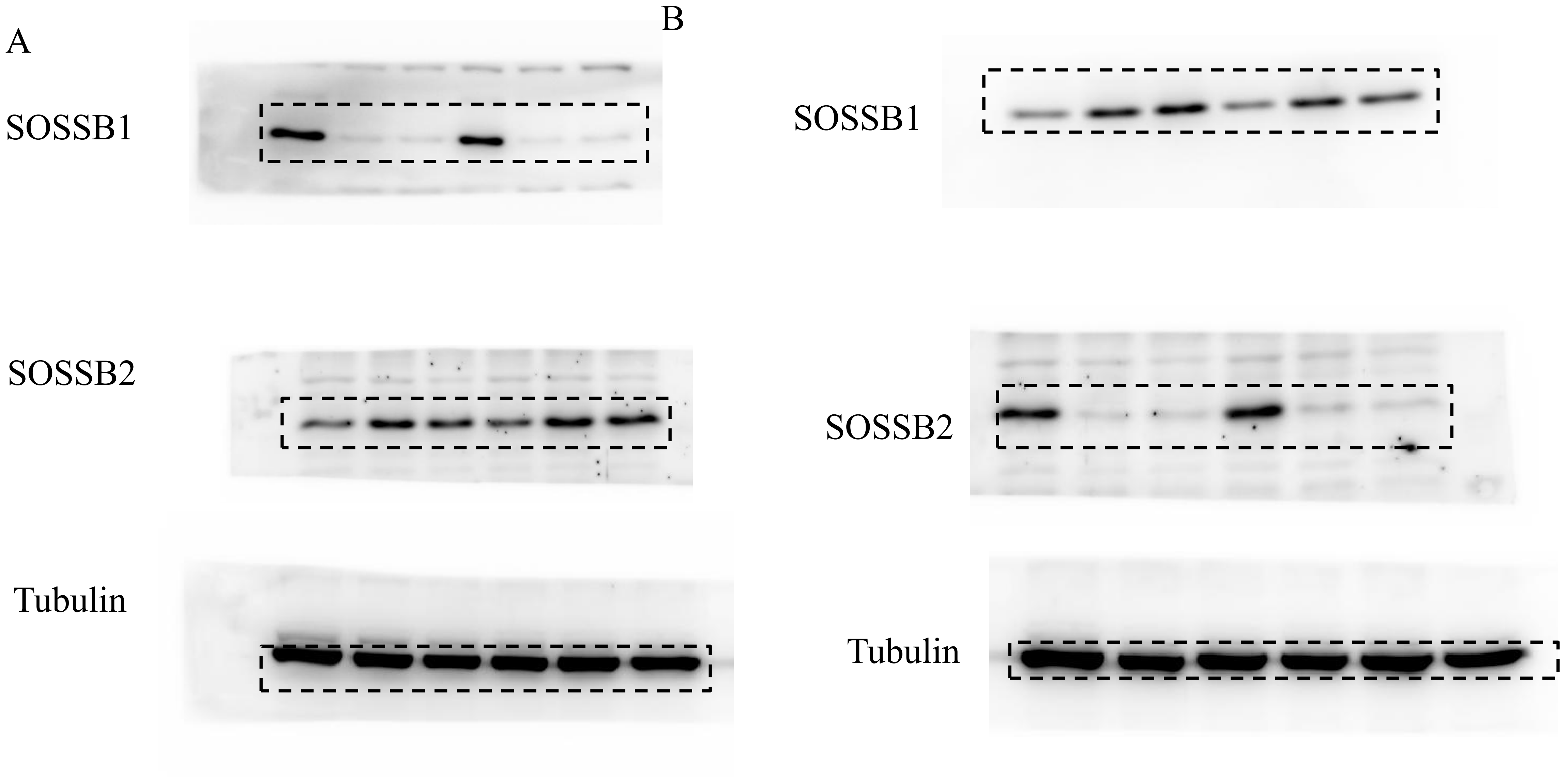

Figure-1

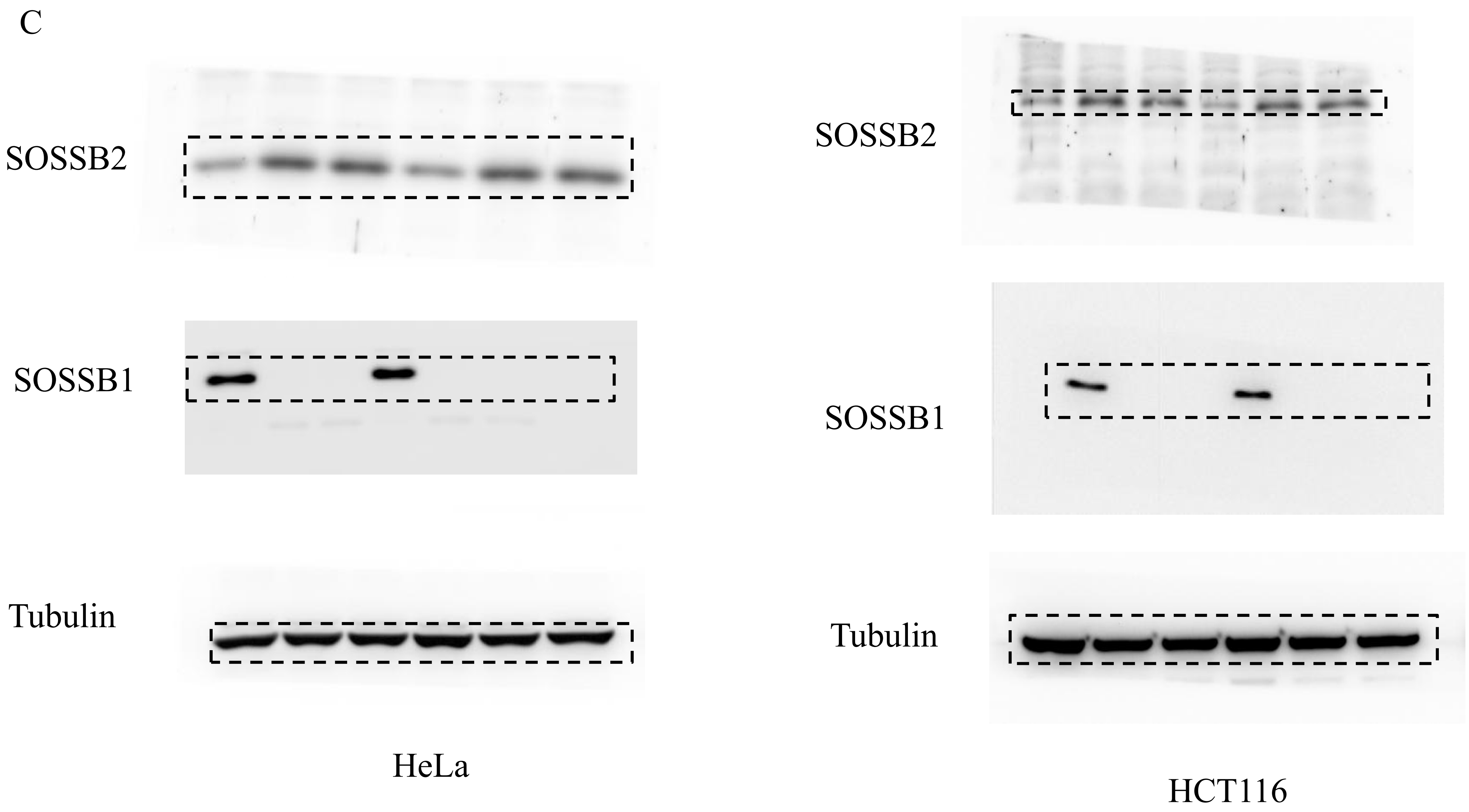

Figure-1

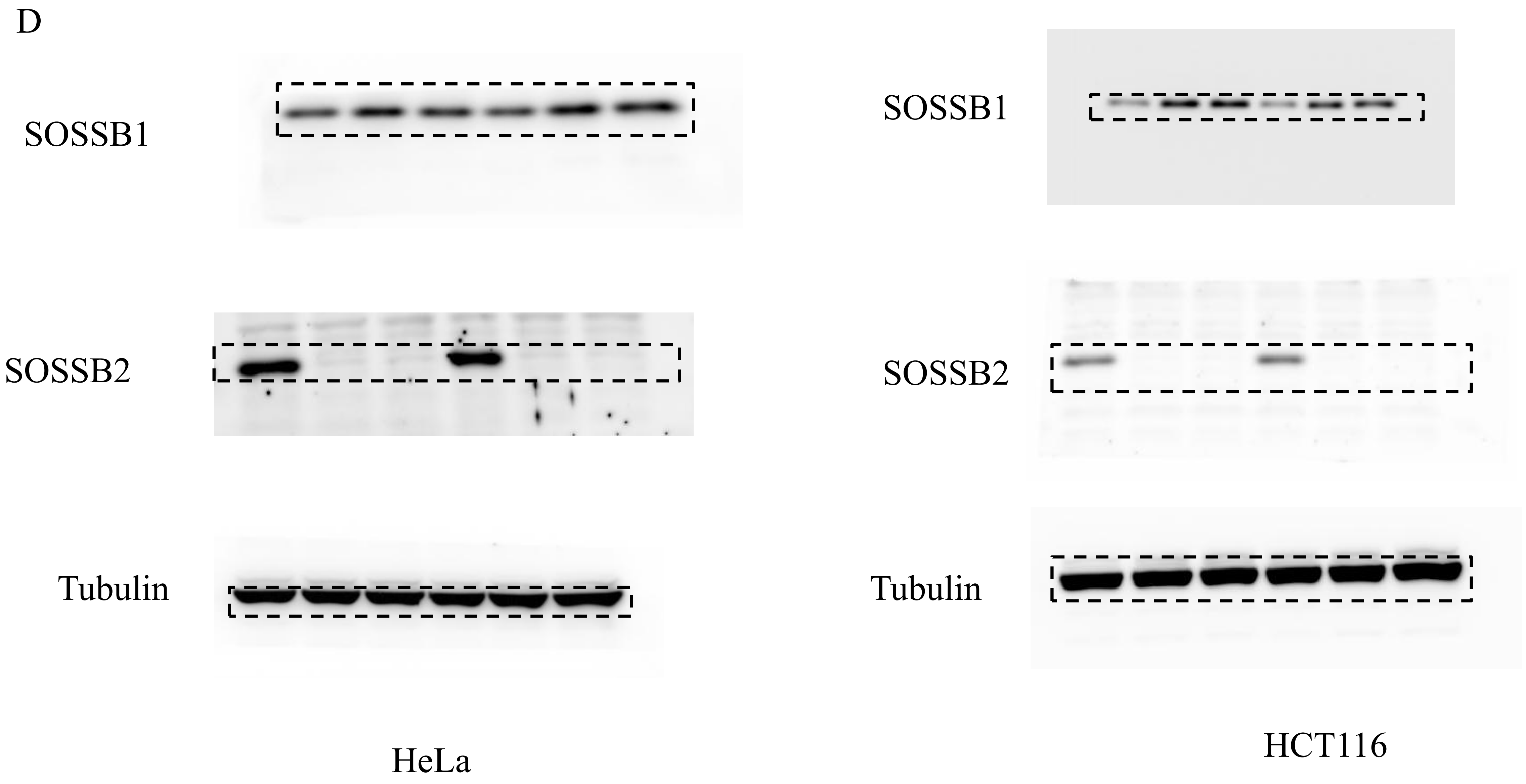

Figure-1

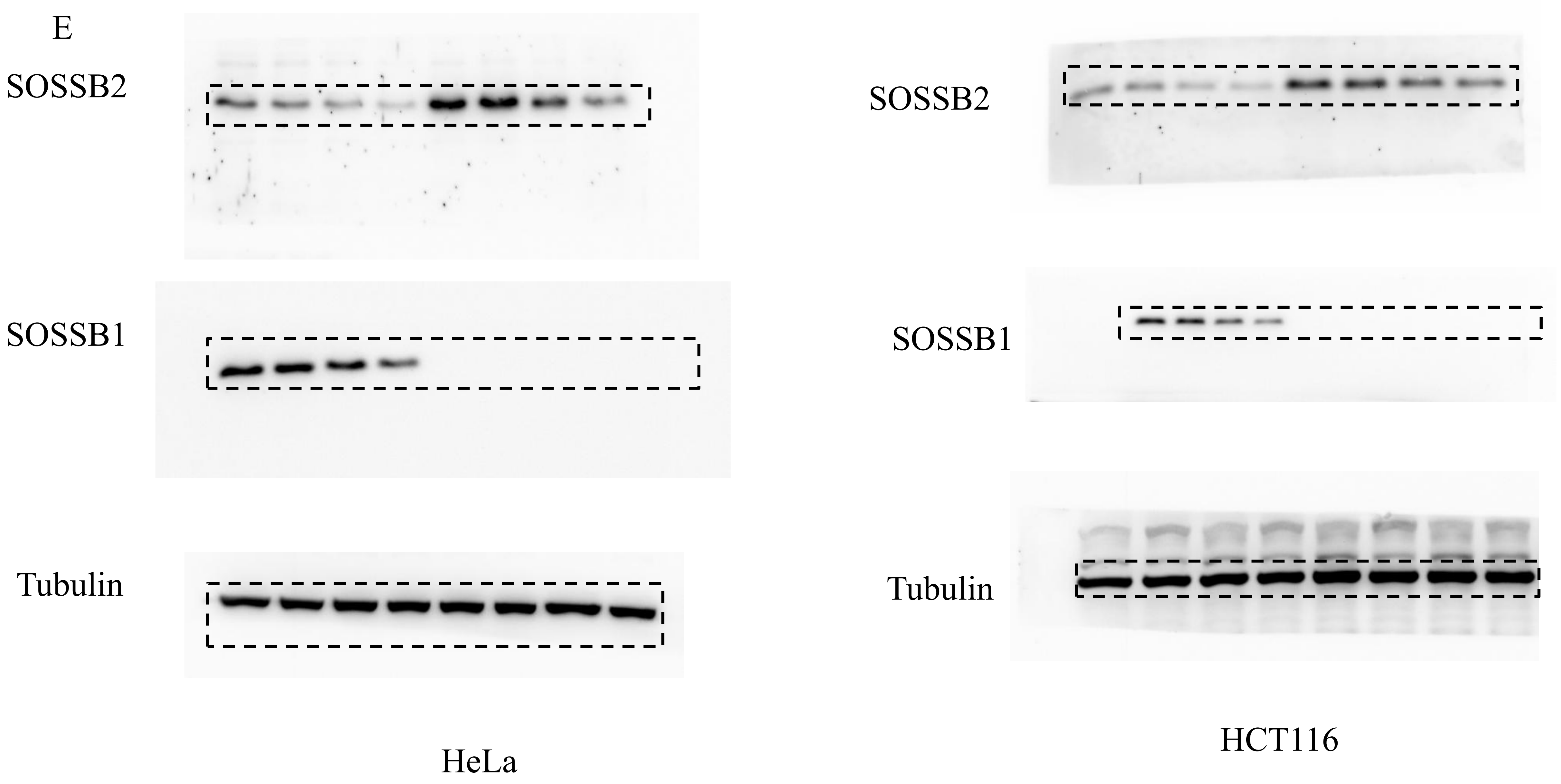

Figure-1

F

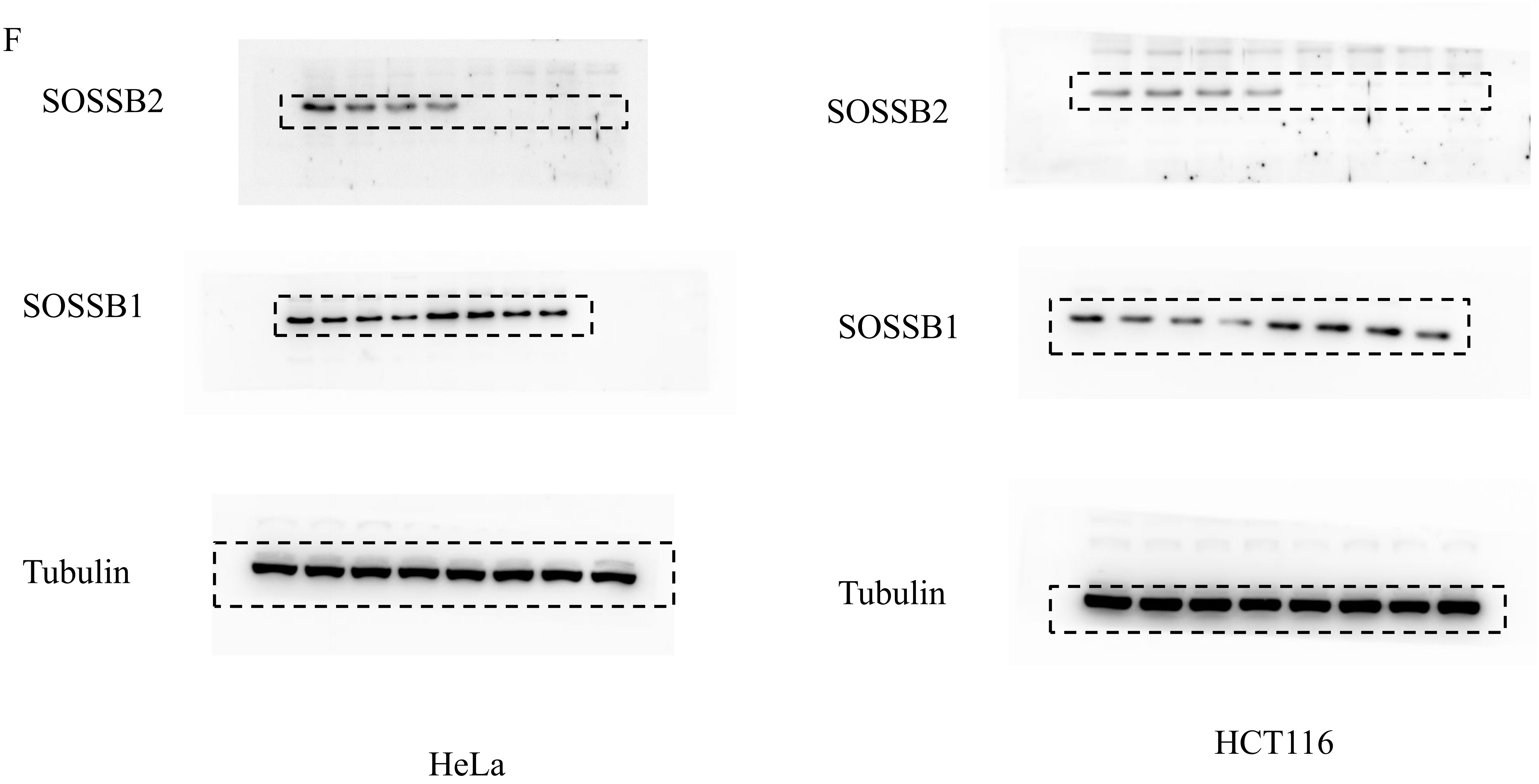

Figure-2 A

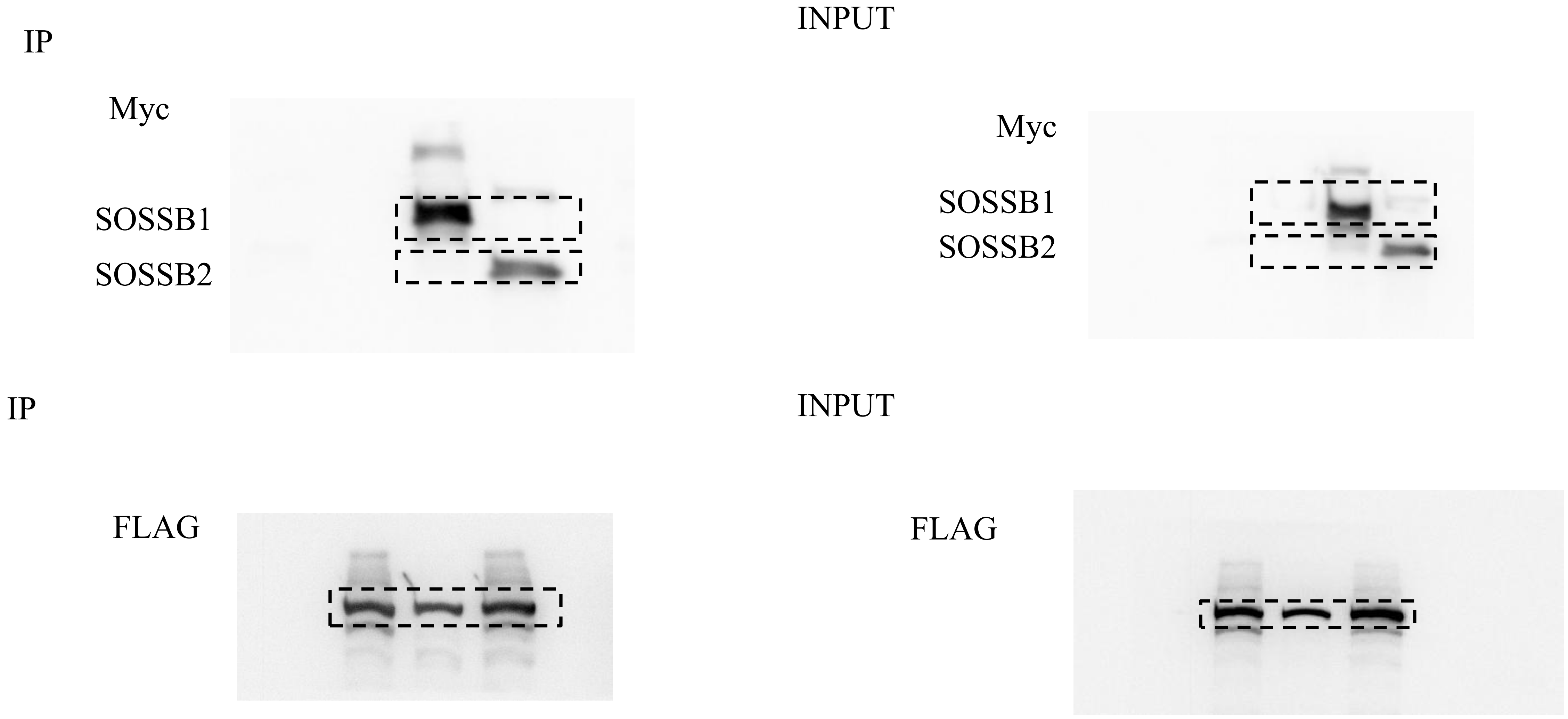

IP
Myc
SOSSB1
SOSSB2
INPUT
Myc
SOSSB1
SOSSB2
IP
FLAG
INPUT
FLAG

Figure-2 B

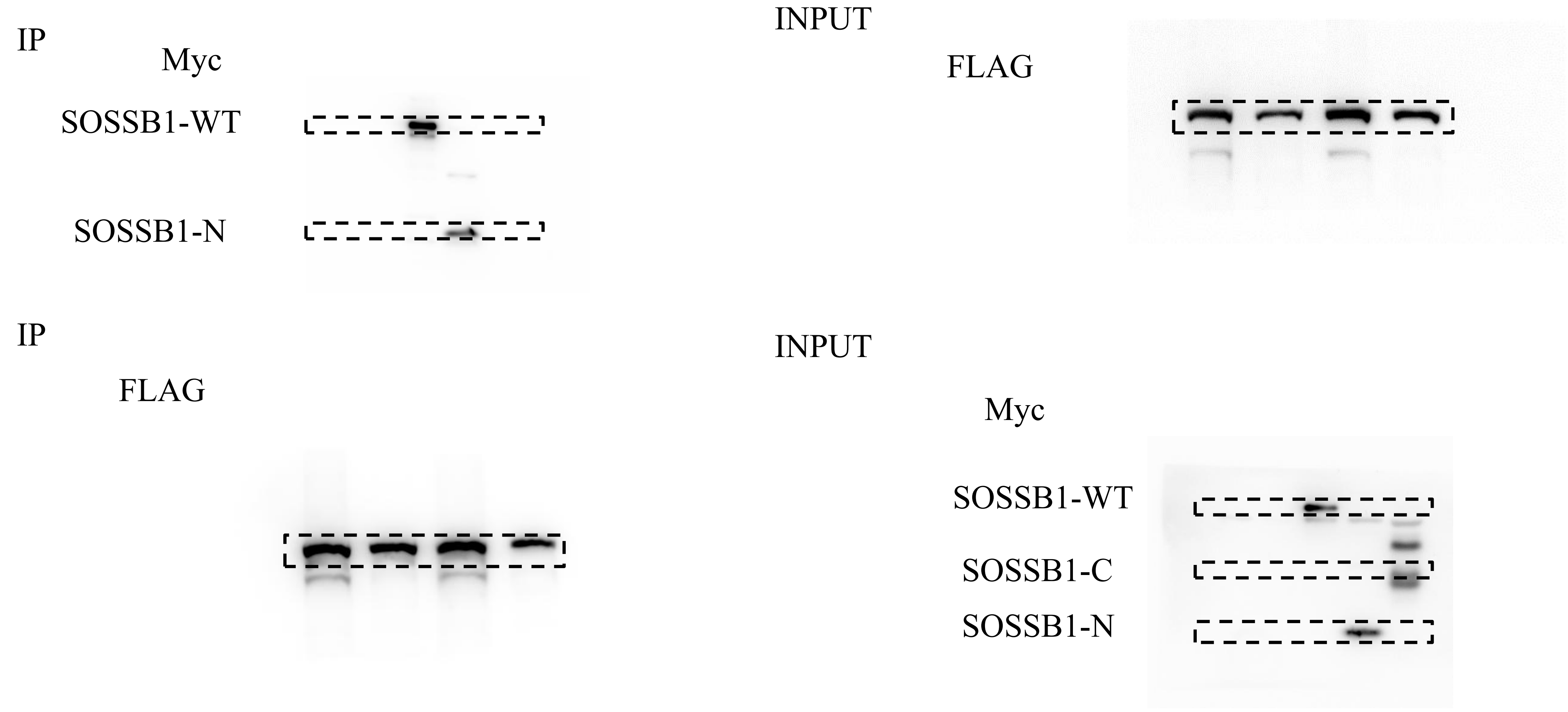
IP
Myc
SOSSB1-WT
SOSSB1-N
INPUT
FLAG
IP
FLAG
INPUT
Myc
SOSSB1-WT
SOSSB1-C
SOSSB1-N

Figure-2 C

IP

Myc

SOSSB2-WT

SOSSB2-N

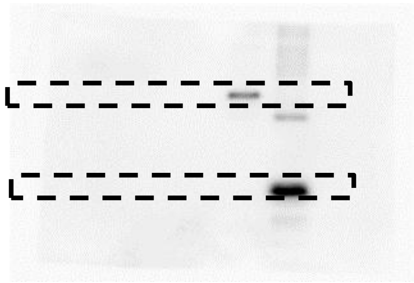

INPUT

FLAG

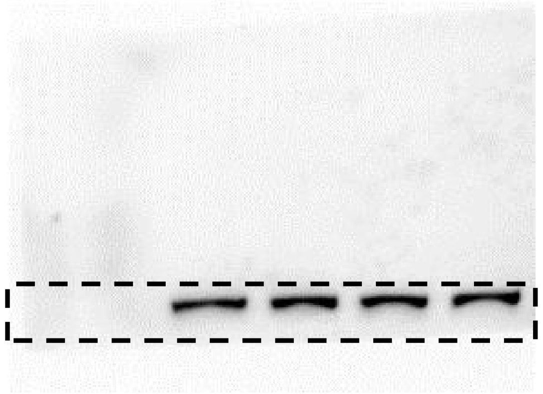

IP

FLAG

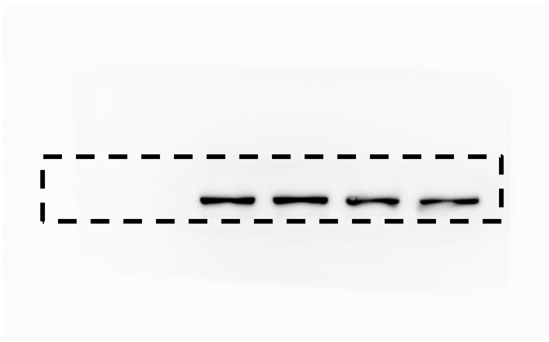

INPUT

Myc

SOSSB2-WT

SOSSB2-C

SOSSB2-N

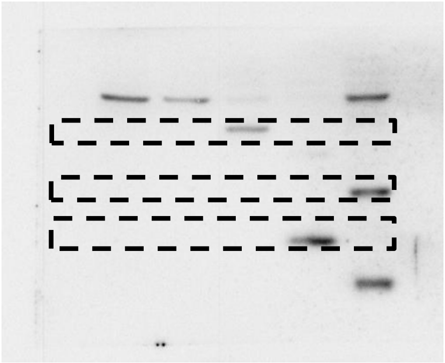

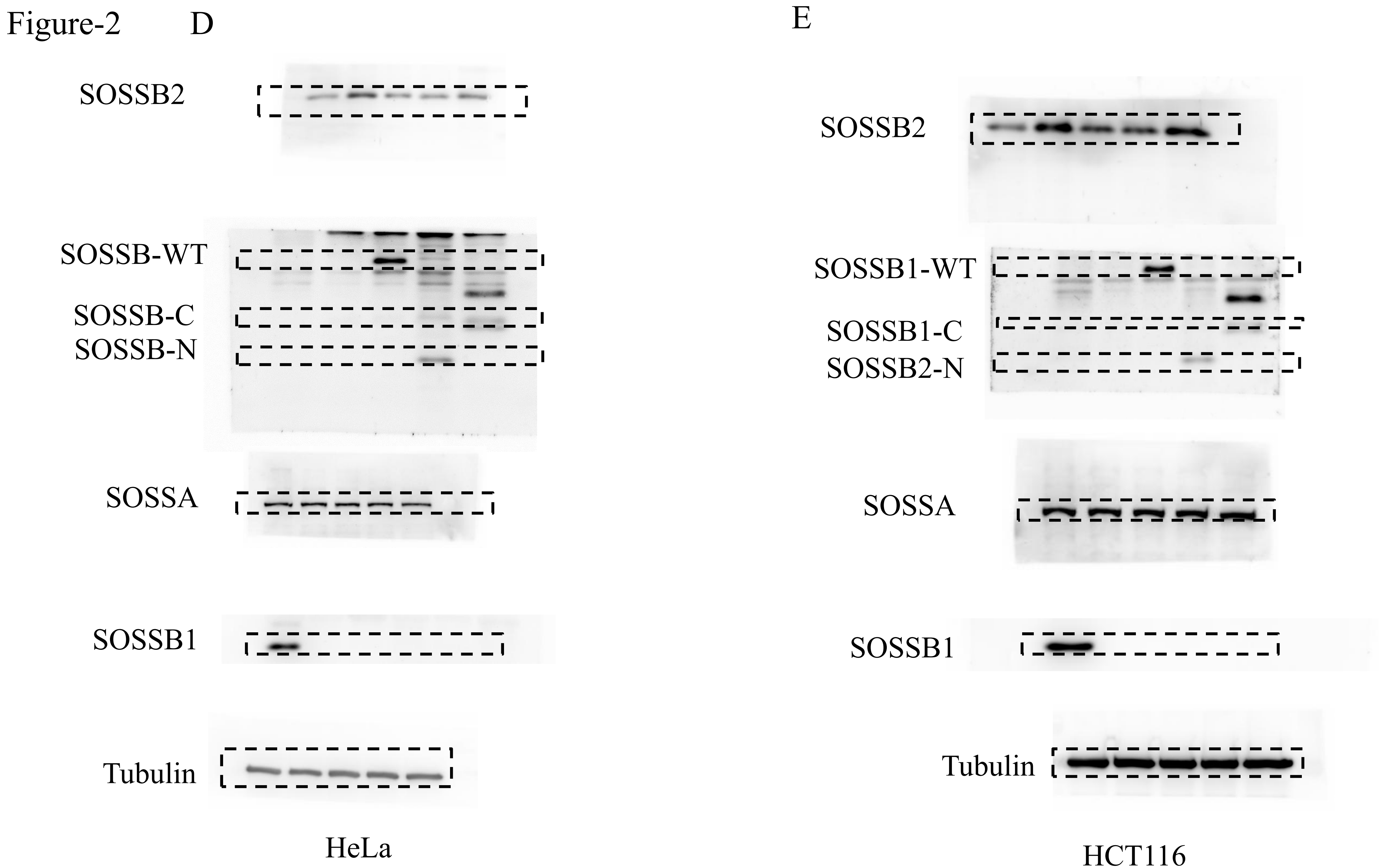
Figure-2
D
E
SOSSB2
SOSSB-WT
SOSSB-C
SOSSB-N
SOSSA
SOSSB1
Tubulin
HeLa
SOSSB2
SOSSB1-WT
SOSSB1-C
SOSSB2-N
SOSSA
SOSSB1
Tubulin
HCT116

Figure-2

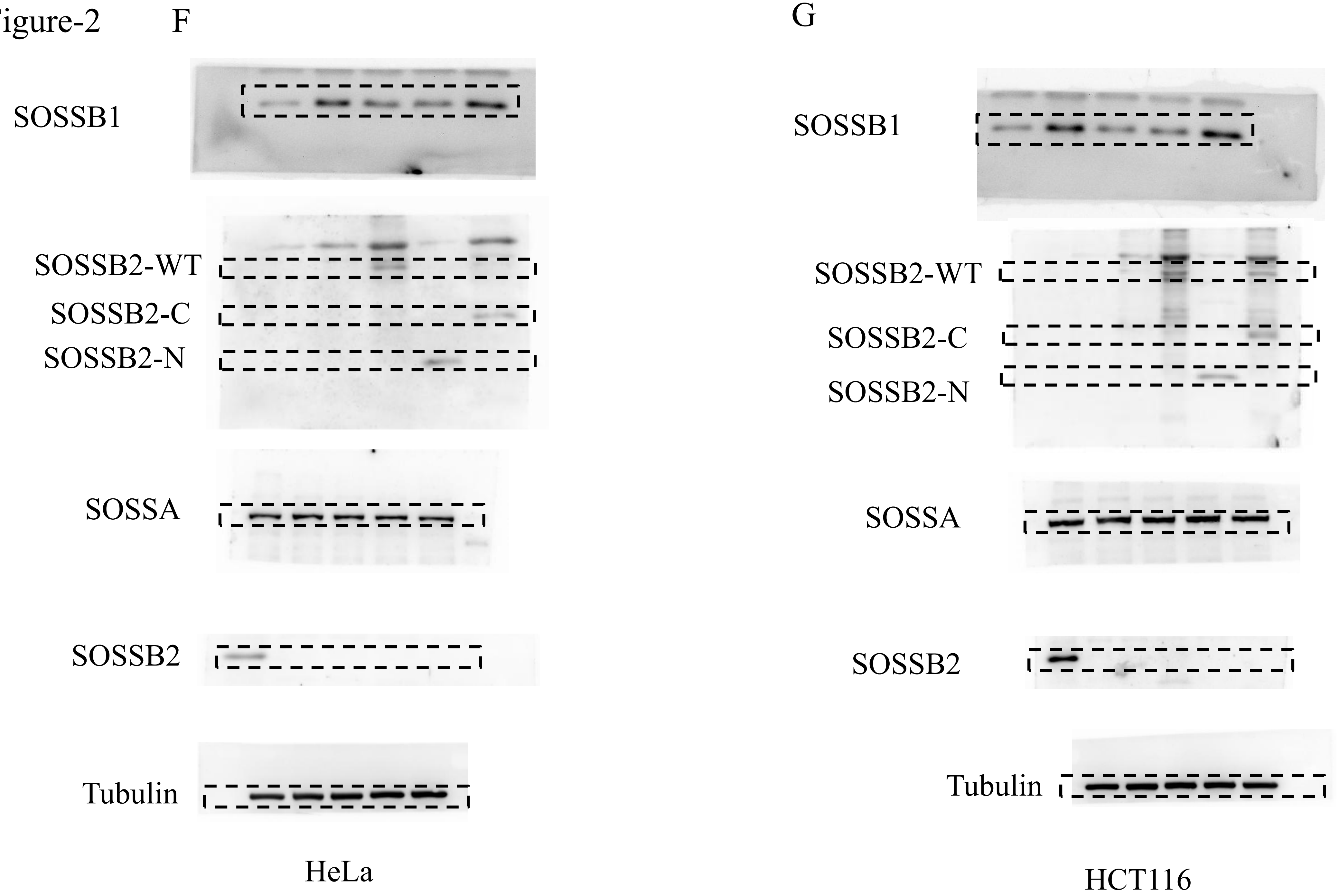
F
SOSSB1
SOSSB2-WT
SOSSB2-C
SOSSB2-N
SOSSA
SOSSB2
Tubulin
HeLa
G
SOSSB1
SOSSB2-WT
SOSSB2-C
SOSSB2-N
SOSSA
SOSSB2
Tubulin
HCT116

Figure-3

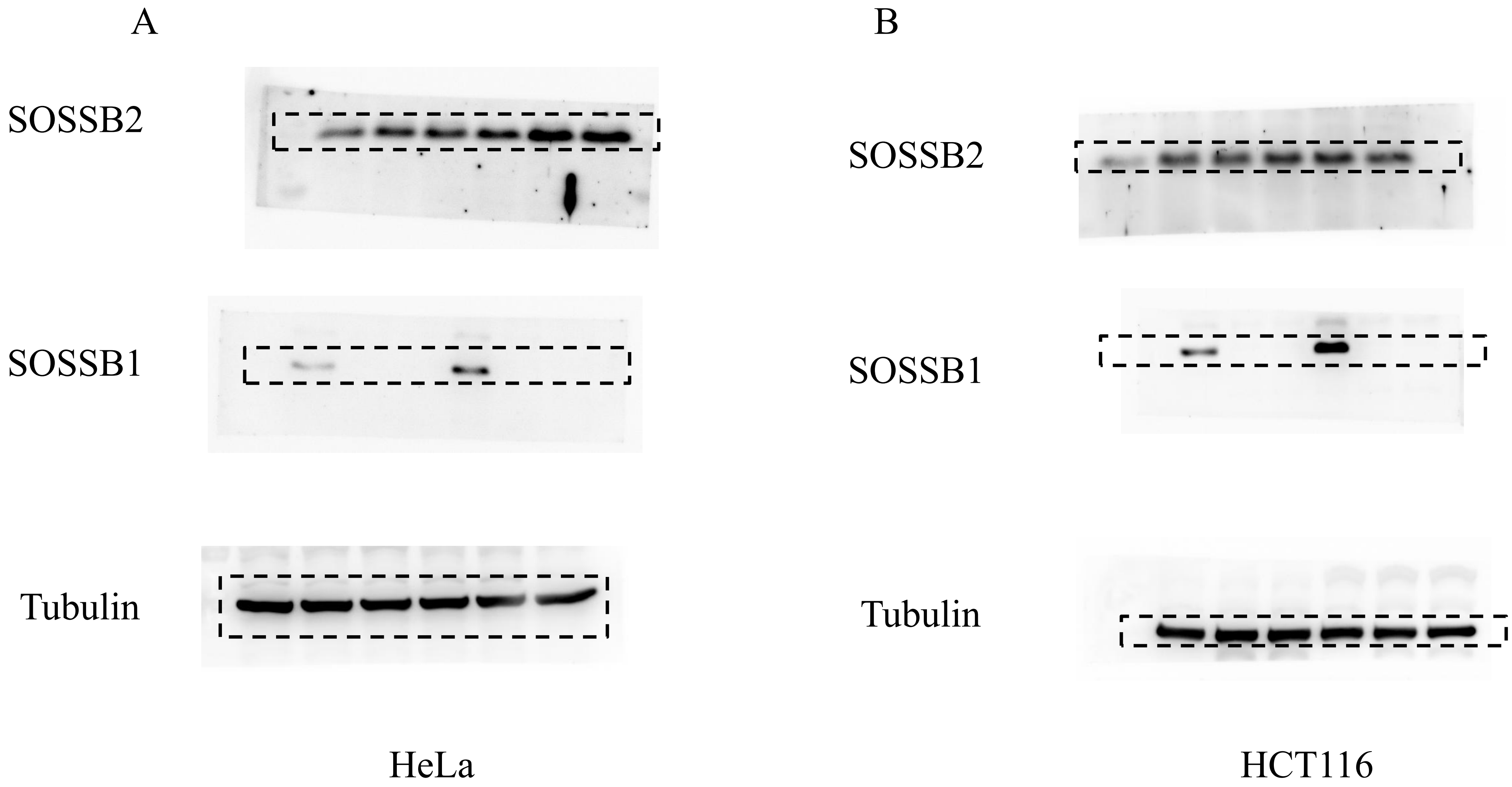
A
B
SOSSB2
SOSSB1
Tubulin
HeLa
SOSSB2
SOSSB1
Tubulin
HCT116

Figure-3

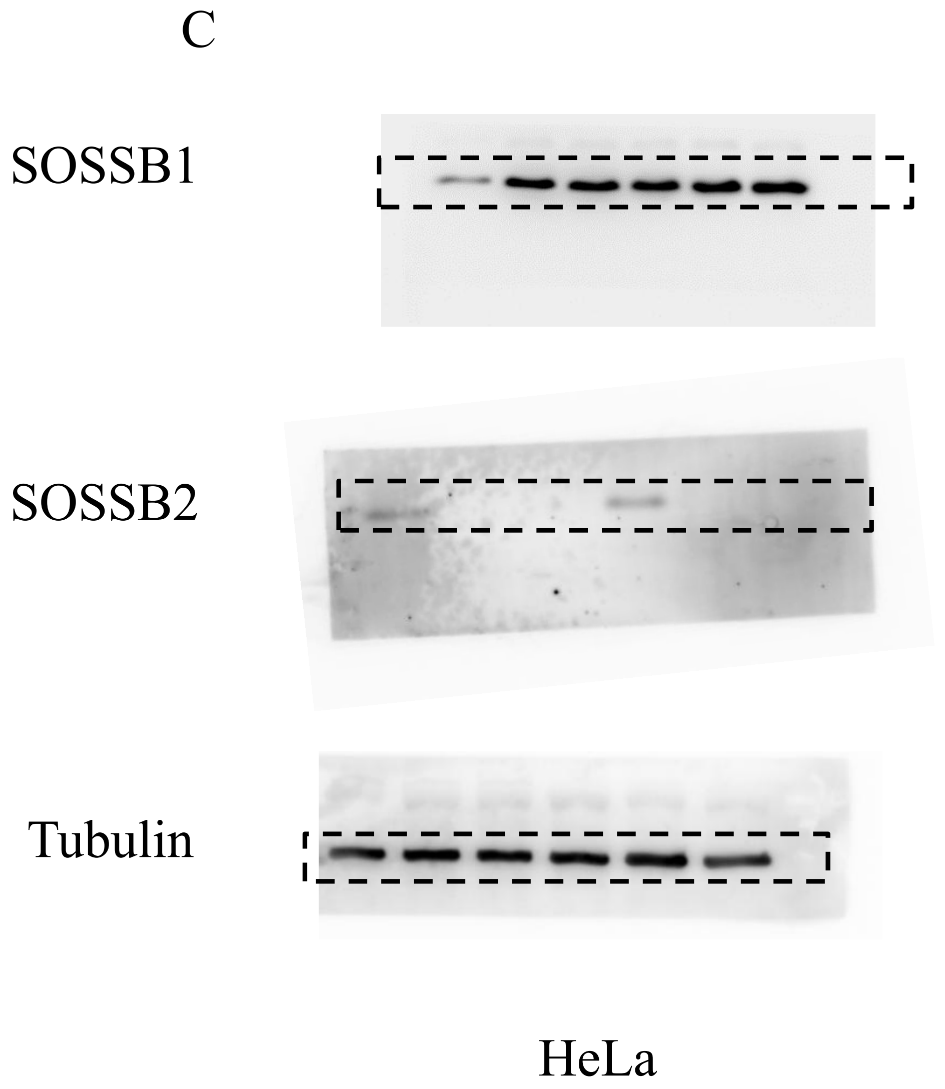
C
SOSSB1
SOSSB2
Tubulin
HeLa

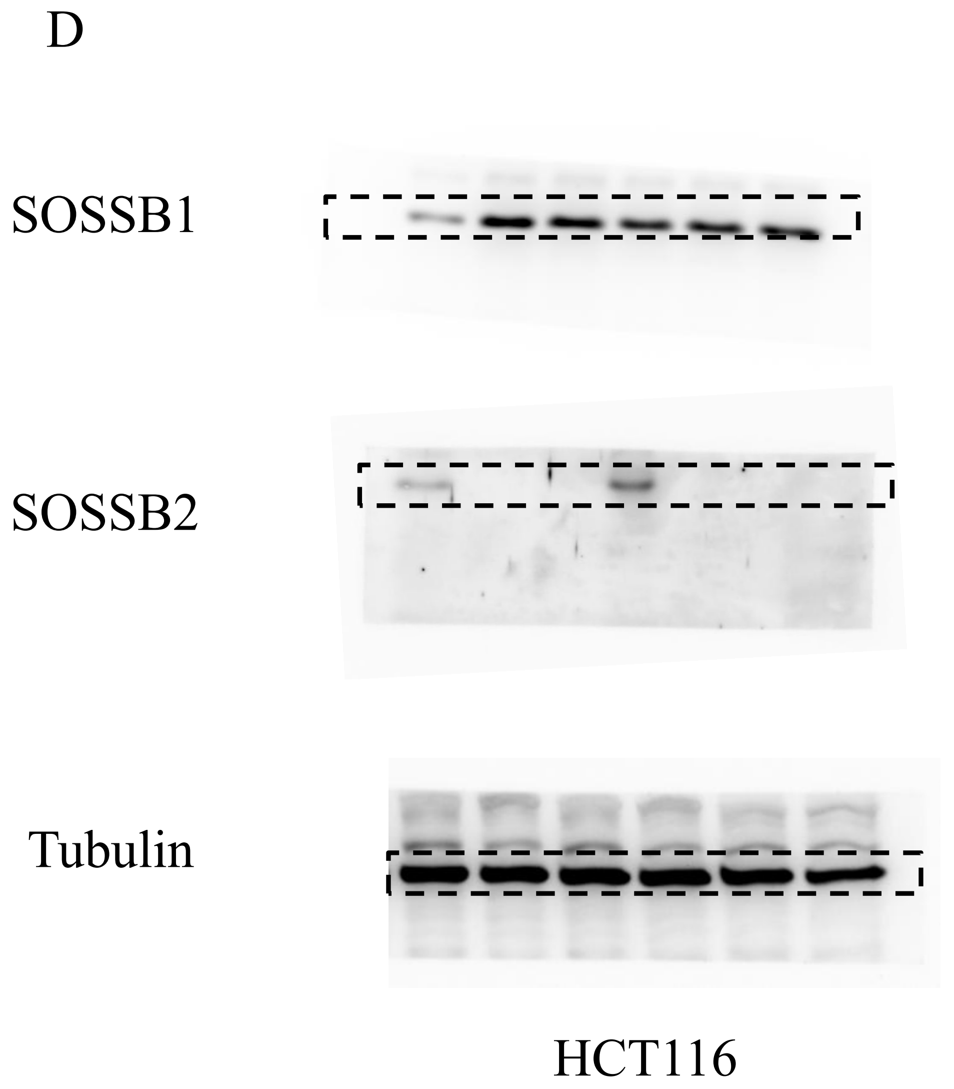
D
SOSSB1
SOSSB2
Tubulin
HCT116

Figure-3

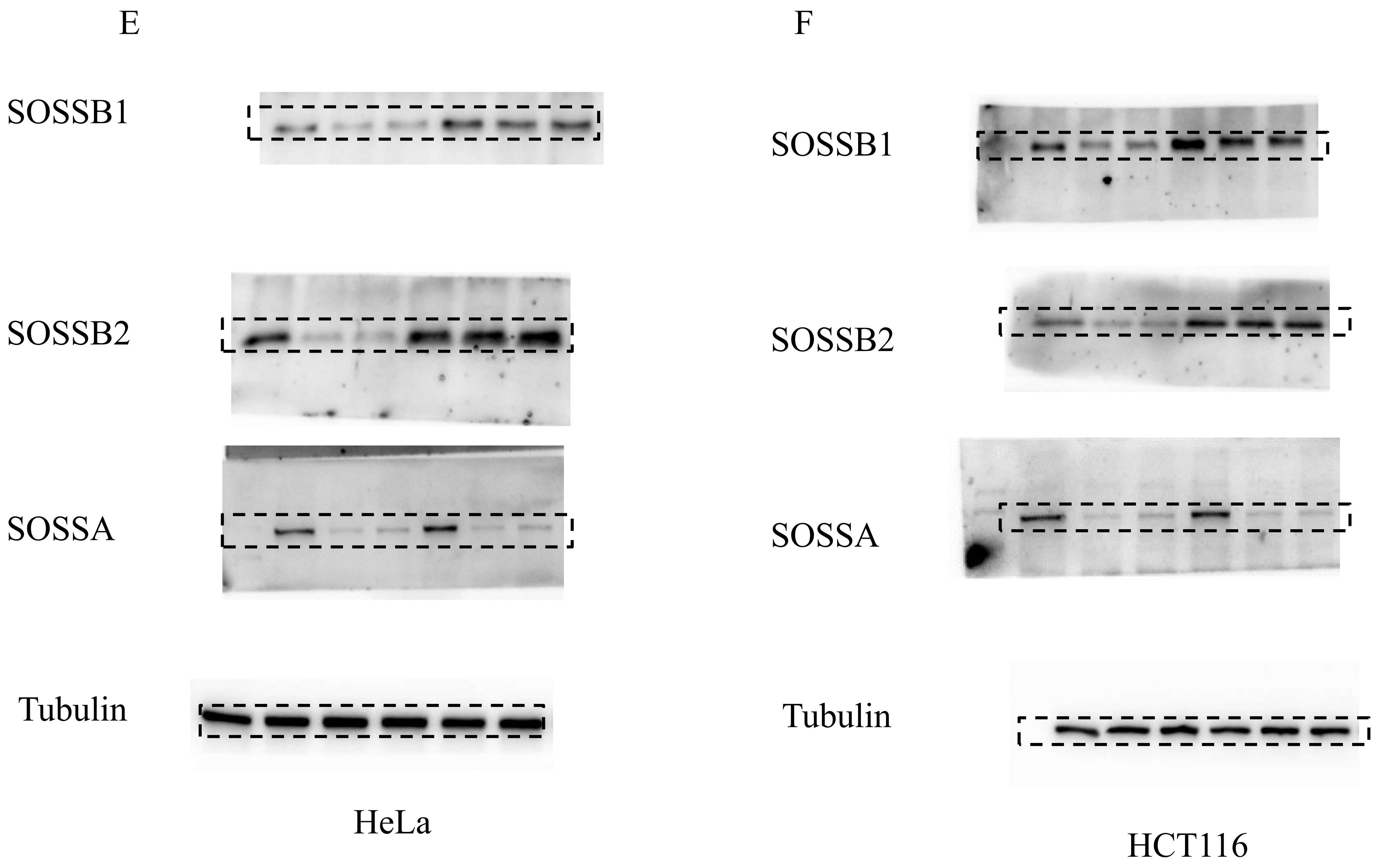
E
F
SOSSB1
SOSSB2
SOSSA
Tubulin
HeLa
SOSSB1
SOSSB2
SOSSA
Tubulin
HCT116

Figure-4

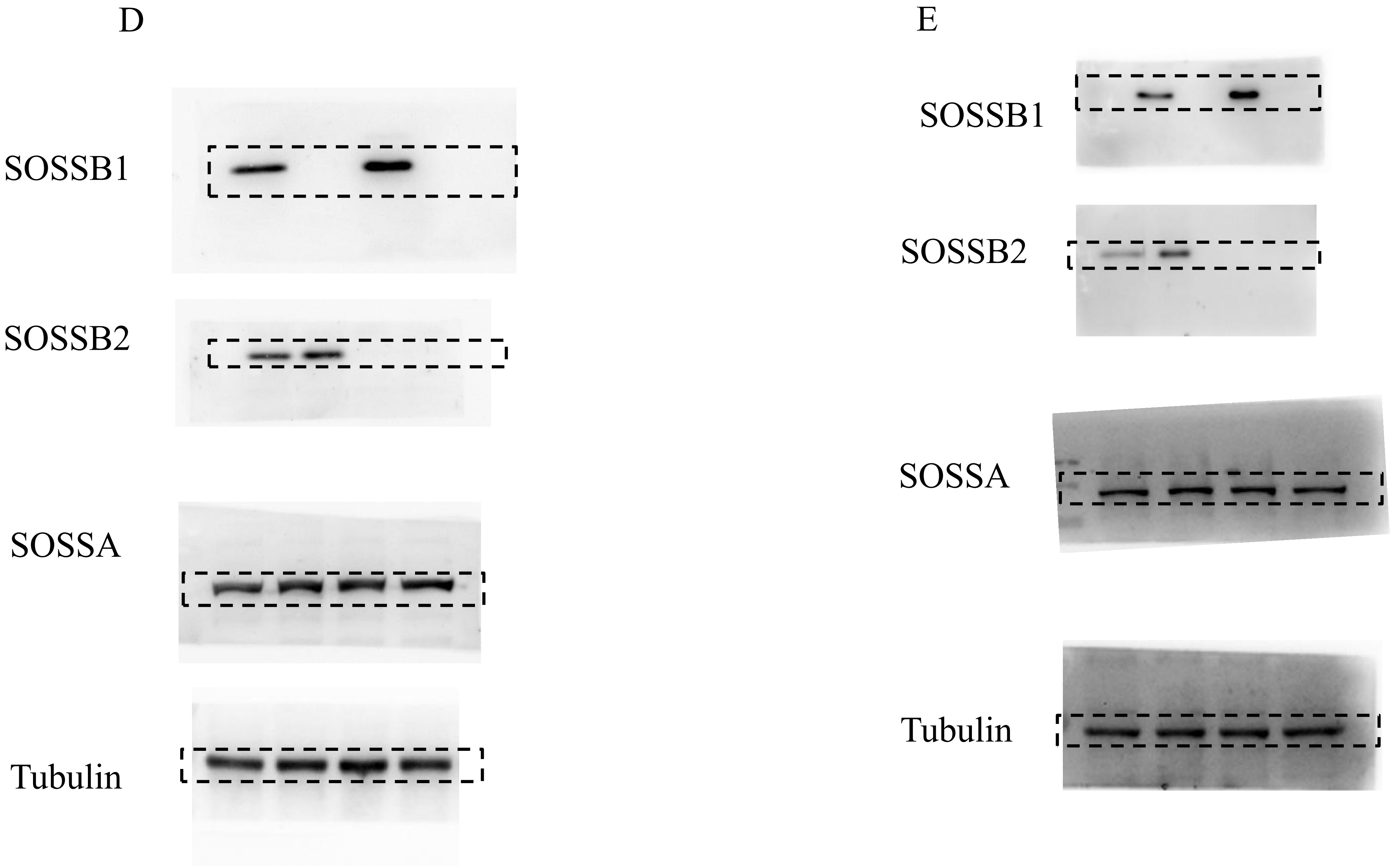
D
SOSSB1
SOSSB2
SOSSA
Tubulin
E
SOSSB1
SOSSB2
SOSSA
Tubulin

Supplementary Figure1

A

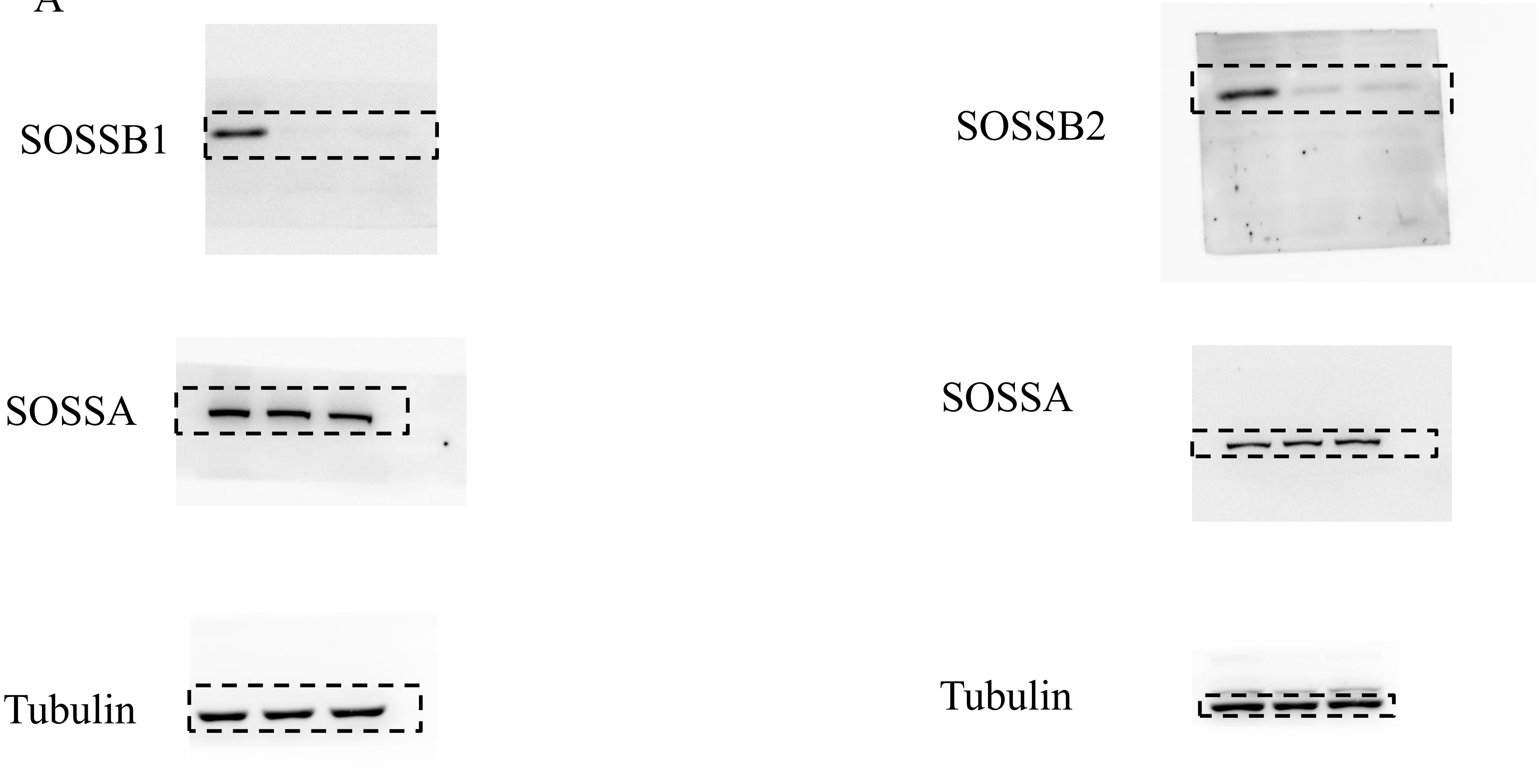

Supplementary Figure 1

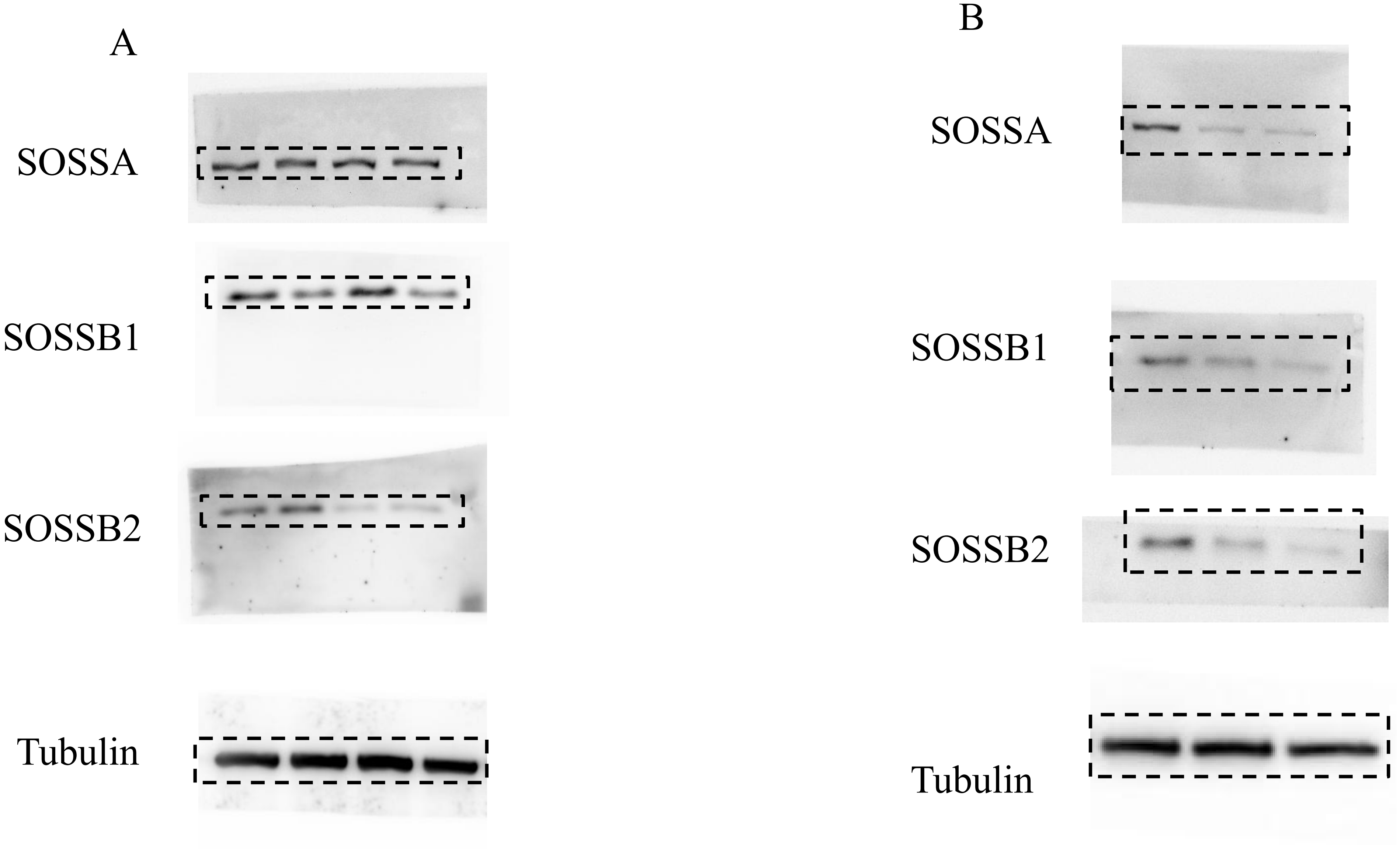

Supplementary Figure 2

A

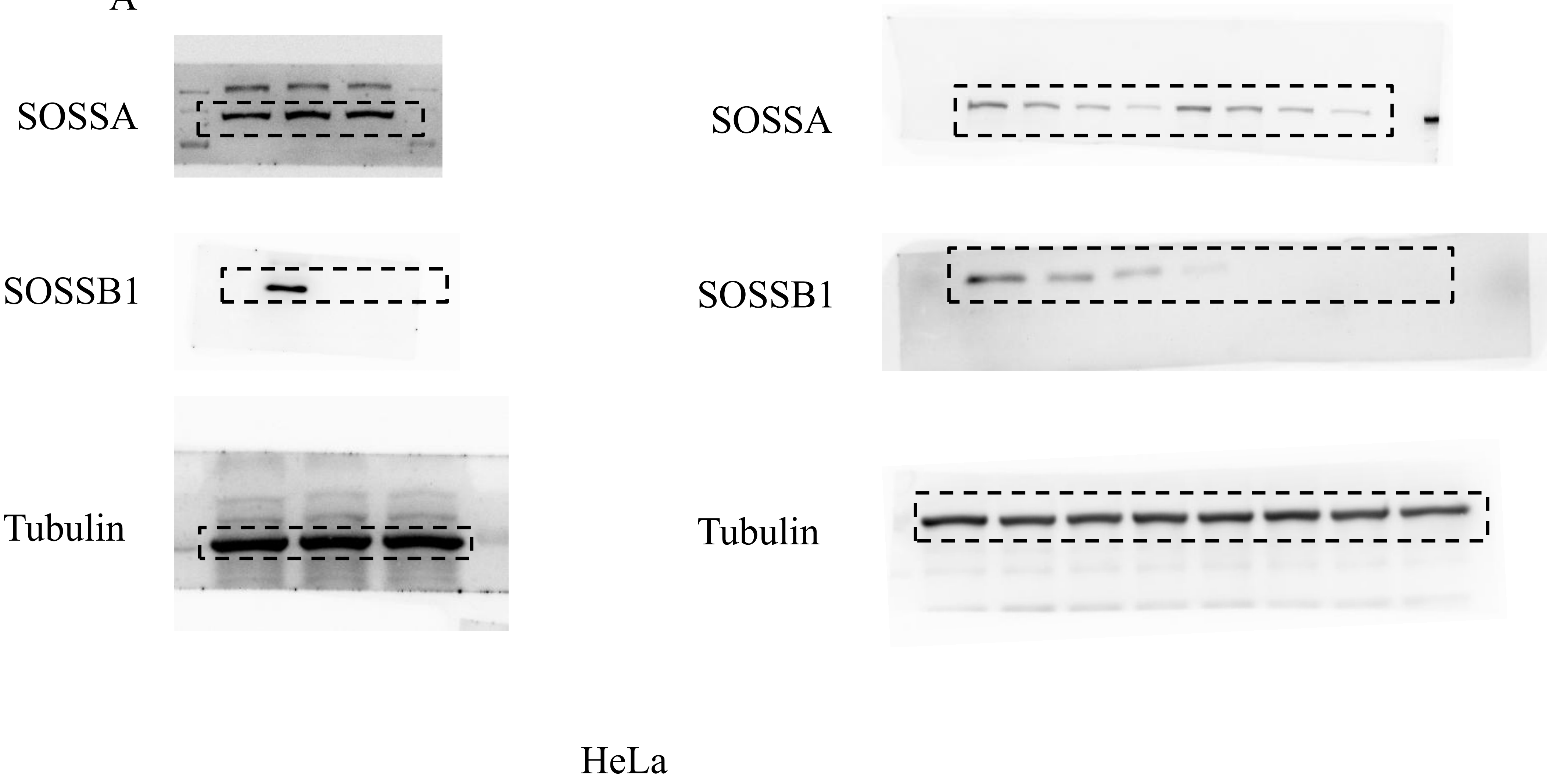

Supplementary Figure 2

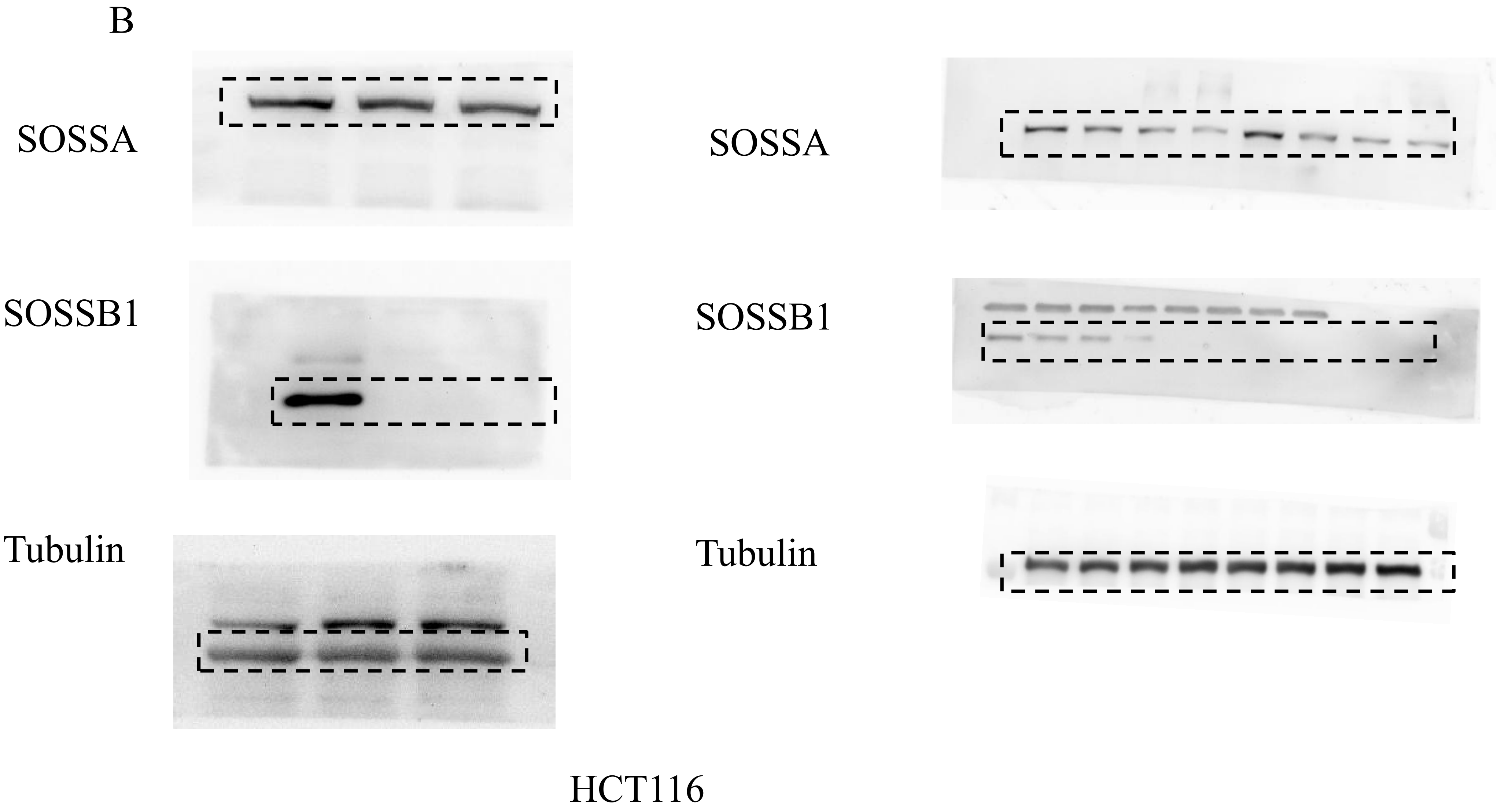

Supplementary Figure 2

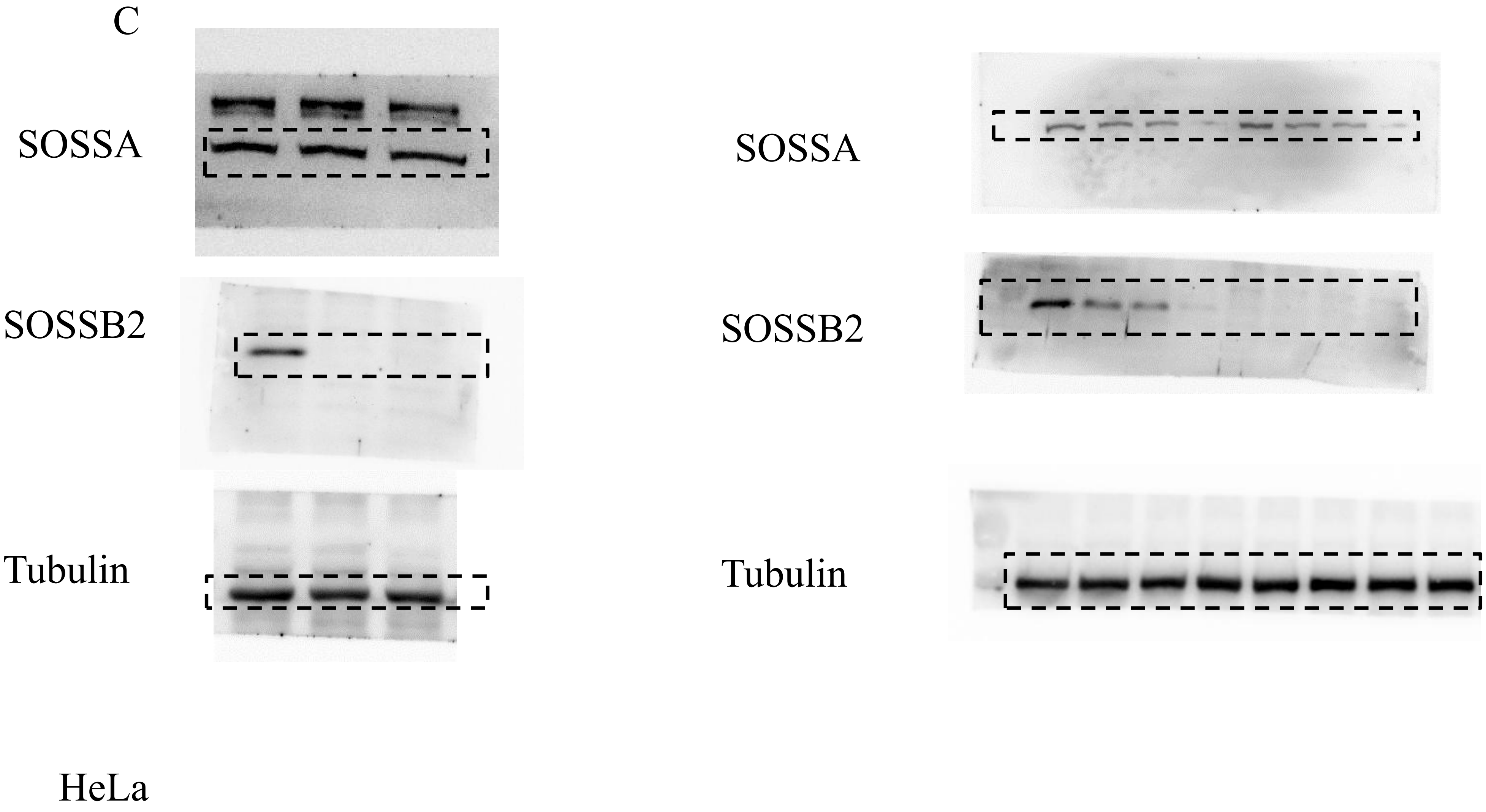

Supplementary Figure 2

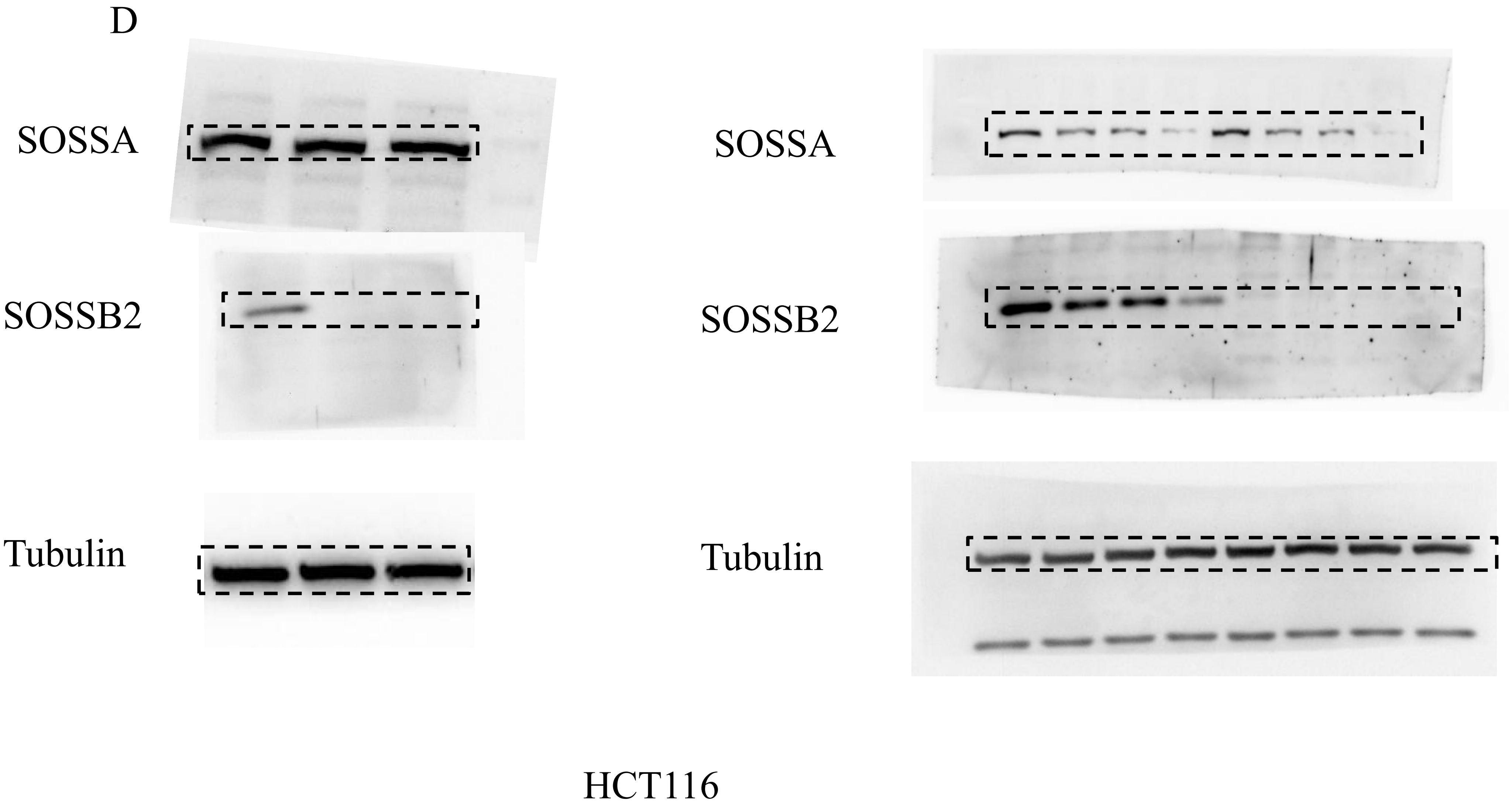

Supplement: Supplementary file 2 — Original western blots [file 41420_2023_1619_MOESM2_ESM.pdf]
